# Loss of *RND3/RHOE* controls entosis through *LAMP1* expression in hepatocellular carcinoma

**DOI:** 10.1038/s41419-024-06420-3

**Published:** 2024-01-13

**Authors:** Sara Basbous, Lydia Dif, Camille Dantzer, Sylvaine Di-Tommaso, Jean-William Dupuy, Paulette Bioulac-Sage, Anne-Aurélie Raymond, Chantal Desdouets, Frédéric Saltel, Violaine Moreau

**Affiliations:** 1 University of Bordeaux, INSERM, BRIC, U1312 Bordeaux, France; 2 CHU de Bordeaux, 33076 Bordeaux, France; 3 Oncoprot Platform, UMS005, TBMCore, University of Bordeaux, 33076 Bordeaux, France; 4 Proteomic plateform, University of Bordeaux, 33076 Bordeaux, France; 5 Sorbonne University, INSERM, Centre de Recherche des Cordeliers (CRC), Paris, France

**Keywords:** Entosis, Cancer

## Abstract

Entosis is a process that leads to the formation of cell-in-cell structures commonly found in cancers. Here, we identified entosis in hepatocellular carcinoma and the loss of Rnd3 (also known as RhoE) as an efficient inducer of this mechanism. We characterized the different stages and the molecular regulators of entosis induced after Rnd3 silencing. We demonstrated that this process depends on the RhoA/ROCK pathway, but not on E-cadherin. The proteomic profiling of entotic cells allowed us to identify LAMP1 as a protein upregulated by Rnd3 silencing and implicated not only in the degradation final stage of entosis, but also in the full mechanism. Moreover, we found a positive correlation between the presence of entotic cells and the metastatic potential of tumors in human patient samples. Altogether, these data suggest the involvement of entosis in liver tumor progression and highlight a new perspective for entosis analysis in medicine research as a novel therapeutic target.

## Introduction

The presence of cell-in-cell (CIC) structures has been observed in a wide range of human cancers and is mainly associated with poor prognosis [1–7]. CIC formation involves a dynamic interaction between an outer and an inner cell. It can be the result of different types of mechanisms involving either homotypic or heterotypic interactions and initiated by either the outer (endocytic CIC) or the inner (invasive CIC) cell [8]. Entosis is defined as a homotypic invasion occurring between tumor cells of the same type where one living cell is internalized inside another one. After internalization by the outer cell, the inner cell is found in the entotic vacuole [9], where it will be degraded generally by autophagy, a non-apoptotic cell death involving the lysosome fusion. Entosis was discovered in vitro in breast cancer cells (MCF-7) cultured under different stress factors such as extracellular matrix detachment, glucose starvation, or ultraviolet radiation [10–12]. Entosis was also described in cells with prolonged aberrant mitosis to eliminate the aneuploid progenies by engulfment, maintaining thus the genome integrity [13]. Mechanistically, entosis was found dependent on cell-cell adhesion molecules such as cadherins and driven by imbalances in actomyosin contractility which is mainly under the control of the RhoA/ROCK pathway [10, 14].

Rnd3, also known as RhoE, is an atypical RhoGTPase protein and a negative regulator of the Rho/ROCK pathway. Others and we previously described Rnd3 as a tumor suppressor in liver cancer [15, 16]. Indeed, *RND3* expression is downregulated in human hepatocellular carcinoma (HCC) samples compared to non-tumoral liver tissues and correlated with poor survival rates [17, 18]. It is implicated in cancer development through the regulation of cell growth and invasion [17, 19]. While working on the role of Rnd3 in HCC, we observed CIC structures, prompting us to study this phenomenon in liver cancer cells. Here, we reported that HCC cells are susceptible to form entosis after Rnd3 downregulation in cultured liver cancer cell lines and in xenografts in mice. Entosis is highly dependent on the RhoA/ROCK pathway, but not on E-cadherin. We also found that Rnd3 loss leads to Lamp-1 up-regulation, required for the internalization and degradation of the entotic cell. Finally, we related that entosis is rare in human HCC tissues, but associated with poor prognosis. Our results suggest that entotic engulfment induced by the loss of Rnd3 in HCC promotes liver tumor progression.

## Results

### Liver cancer cells are prone to perform entosis

**Fig. 1:**
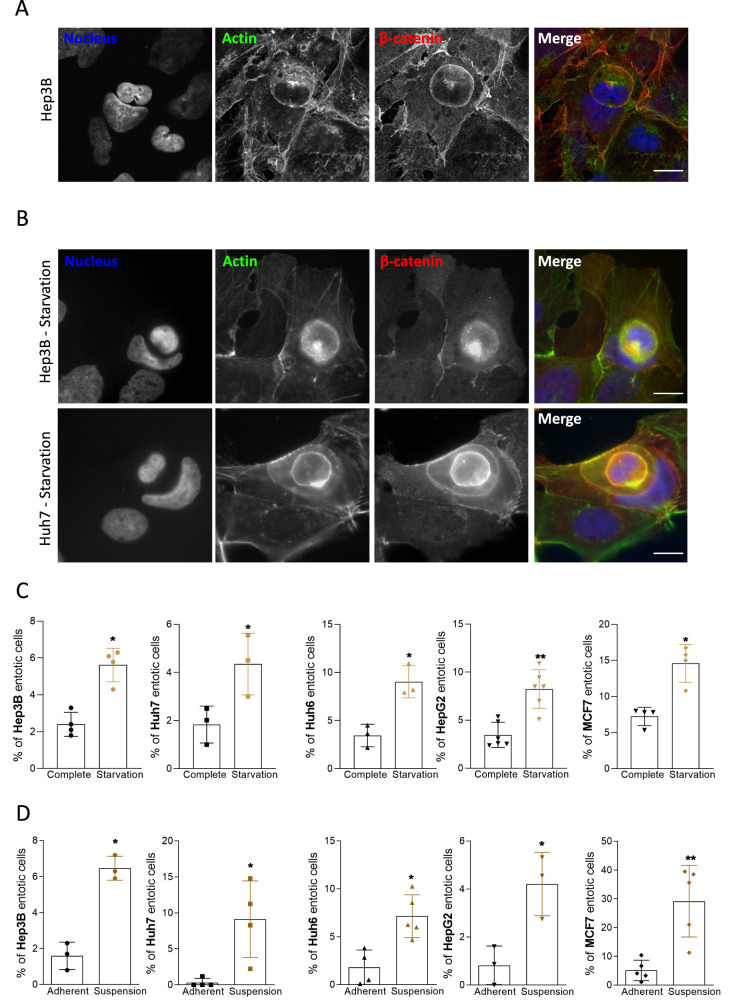
Liver cancer cells perform entosis upon starvation and matrix detachment. **A** Representative Hep3B entotic cell characterized using confocal microscopy by the nucleus deformation showed with the DAPI staining (blue) and high concentration of actin with the phalloidin (green). Beta-catenin staining (red) was used to confirm that the inner cell is inside the outer cell. Scale bar, 15 µm. **B** Nutrient starvation induces entosis as determined by immunofluorescence in Hep3B and Huh7 cells stained for nucleus (blue), actin (green), and beta-catenin (red). Scale bar, 15 µm. **C** Graphs show the quantification of entosis induced by starvation in Hep3B, Huh7, Huh6, and HepG2 cells. **D** Matrix detachment favors entosis in liver cancer cells. Hep3B, Huh7, Huh6, and HepG2 cells were cultured in non-adherent conditions and analyzed for their ability to form entosis. Graphs show the quantification of entosis induced by suspension culture conditions. **B**–**D** MCF-7 breast cancer cells were used as a positive control. Error bars: SD of three or more independent experiments. Significance was determined with the Mann–Whitney *U*-test.

In order to investigate entosis in liver cancer, cultured cells (Hep3B, Huh7, Huh6, or HepG2) were subjected to stress conditions known to favor entosis in breast cancer cells such as nutrient deprivation [11] or matrix detachment [10]. MCF-7 human breast cancer cells were used as a positive control. Cell internalization was analyzed and quantified by confocal microscopy after immunostaining of nuclei, F-actin, and β-catenin to visualize nucleus deformation, shape, and cell periphery respectively. The outer cell shows a crescent-shaped nucleus, whereas the inner cell is rounded and retains its plasma membrane (Fig. 1A). In normal conditions, entotic structures can be observed in about 1–3% of total adherent liver cancer cells. After nutrient deprivation, a significant increase in the percentage of entotic cells is observed in all four cell lines, with up to 10% of entotic cells in HepG2 and Huh6 cells, when compared to normal culture conditions (Fig. 1B–C). Matrix detachment is also described as an inducer of entosis in MCF-7 cells [10]. In order to test this stimulus, cells were cultured in non-adherent conditions and deposited on a slide by cytospin for immunostaining. The results show an increase of entotic cells after matrix detachment in all liver cancer cells, even if the percentage of entotic cells remains lower in liver cancer cells (6–14%) when compared to MCF-7 cells (Fig. 1D).

As entosis is favored under stress conditions, we attempted to address whether hypoxia or drug treatment may trigger this mechanism in liver cancer cells. Hypoxic conditions were monitored by the increase in the mRNA expression of *GLUT1* and *VEGF*, two target genes of Hypoxia-Inducible Factor (Hif1α) [20] (Supplementary Fig. 1A). The quantification of entotic Hep3B and Huh7 cells under hypoxia did not show any significant change compared to basal levels (Supplementary Fig. 1B). We also evaluated whether HCC treatments like Sorafenib could be an inducer of entosis. While Sorafenib treatment altered pERK/ERK ratio as expected, no significant difference was observed in the percentage of entosis in treated versus untreated HCC cells (Supplementary Fig. 1C). Altogether, our results demonstrate that liver cancer cells are prone to perform entosis upon proper stimuli such as nutrient deprivation or matrix detachment.

### CIC formation is driven by the loss of Rnd3 expression

**Fig. 2:**
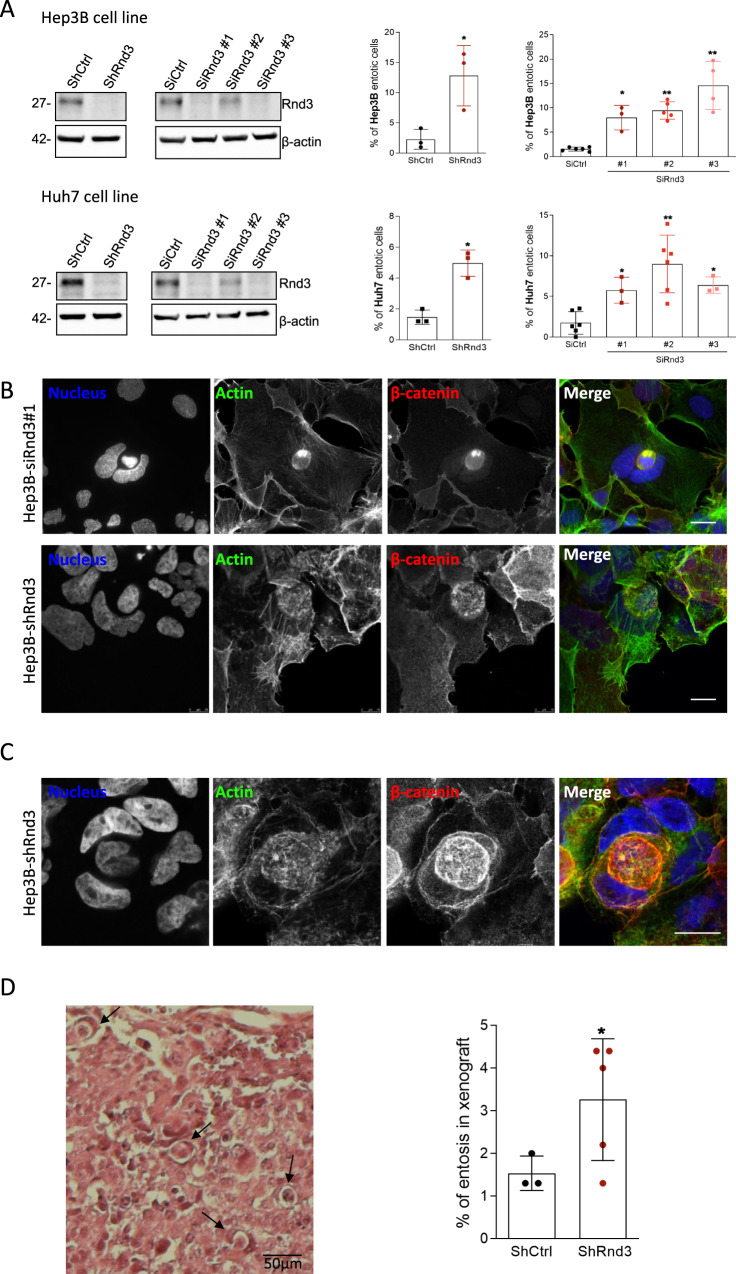
Rnd3 silencing promotes entosis in hepatocellular carcinoma cells. **A** The inhibition of Rnd3 expression was performed in Hep3B (upper panel) and in Huh7 (lower panel) cells using either a shRNA targeting Rnd3 (ShRnd3) inducible with doxycycline treatment and compared to a control shRNA (ShCtrl) or three independent siRNA targeting Rnd3 SiRnd3 #1, SiRnd3 #2, SiRnd3 #3 and SiCtrl as control. Rnd3 knock-down was assessed by Western blot, β-actin is the loading control. Graphs show the quantification of entotic cells upon Rnd3 KD. Error bars: SD of three or more independent experiments. Significance was determined with the Mann–Whitney *U*-test. **B**, **C** Representative of a single (**B**) or a double (**C**) event of entosis after silencing of Rnd3 expression in Hep3B cells with siRnd3 (**B**, upper panel) or with shRnd3 (**B**, lower panel; and **C**). Cells were labeled with DAPI, phalloidin, and beta-catenin antibody to visualize the nucleus, actin, and membrane, respectively. Scale bar, 15 µm. **D** Entosis was visualized in fixed tumor tissues from mice subcutaneously inoculated with Hep3B-shCtrl (*n* = 3) or Hep3B-shRnd3 (*n* = 5) cells. Tumor sections were stained with hematoxylin-eosin (HE) and the percentage of entotic cells was quantified. Scale bar, 50 µm. Error bars: SD of three or more independent samples. Significance was determined with the Mann–Whitney *U*-test.

While working on the role of Rnd3 in HCC progression [17, 19], we noticed that, upon Rnd3 silencing, some cells appeared to be inside other cells, reminiscent of entosis. To examine whether the silencing of Rnd3 may trigger this mechanism, we first analyzed Rnd3 expression in liver cancer and MCF-7 cells. While Hep3B and Huh7 cells strongly express Rnd3 and Huh6 cells slightly less, Rnd3 is not expressed in HepG2 or MCF-7 cells (Supplementary Fig. 2A). We thus choose to silence Rnd3 in Hep3B and Huh7 cells using two different approaches: transient transfection of siRNAs using three different siRNAs targeting Rnd3 (SiRnd3#1, #2, #3), or inducible Rnd3 knockdown (KD) in cell lines stably expressing shRNA [19]. We found that Rnd3 KD led to a significant increase of CIC events in Hep3B and Huh7 cells regardless of the approach used (Fig. 2A). Neighboring cells engulfed each other, with about 10% of adherent cells containing engulfed neighbors. CIC events induced by the loss of Rnd3 show similar characteristics to stress-induced entotic cells, such as crescent-shaped nuclei and round-engulfed cells (Fig. 2B). More complex cell structures were observed, with three or more cells involved in sequential engulfments (Fig. 2C). Thus, Rnd3 loss-mediated CIC events share common features with entosis observed in nutrient-depleted or matrix-detached conditions. Moreover, the combination of stimuli, i.e., silencing of Rnd3 and starvation did not increase the entosis mechanism in Hep3B and Huh7 cells, suggesting the involvement of the same pathways to mediate cell engulfment (Supplementary Fig. 2B, C). The loss of Rnd3 expression, and therefore function, appears to be a trigger of cell engulfment in HCC cells. We next aimed to identify entosis in vivo in tumor tissues using the xenograft mouse model described previously [19]. We noticed a significant increase in entosis percentage in tumors generated from Rnd3-KD cells when compared to control Hep3B cells (Fig. 2D). Thus, Rnd3 loss favors HCC CIC events both in vitro and in vivo.

### Characteristics of entosis stages after silencing of Rnd3

**Fig. 3:**
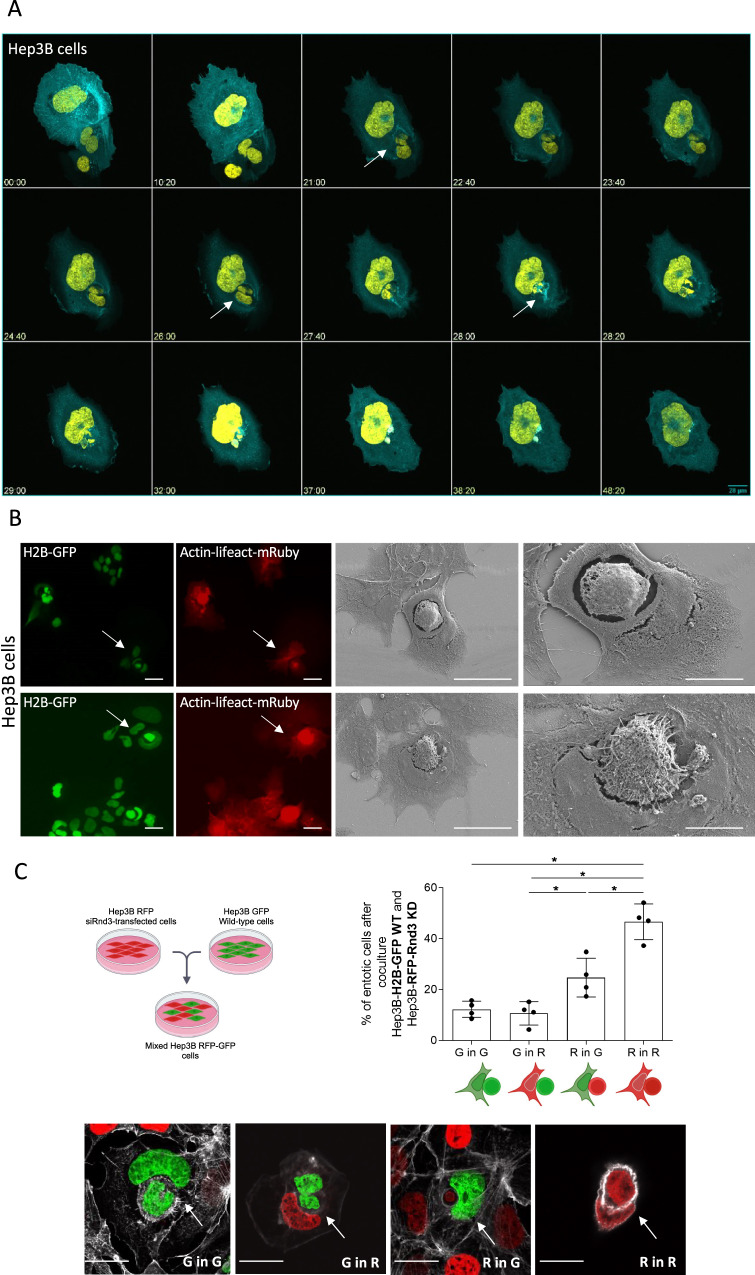
Characterization of entosis mediated by the loss of Rnd3 expression. **A** Dynamics of entosis upon Rnd3 silencing. Hep3B cell lines were transduced with H2B-GFP (yellow) and LifeAct-mRuby (cyan) to mark the nucleus and the actin, respectively. Cells were transfected with siRNA targeting Rnd3 and spinning-disk microscopy analysis was done over 48 h. The gallery corresponds to Video 1. Time is in hours. Three stages were defined: (i) cell contact, (ii) internalization, arrows show the internalization of the inner binuclear cell 21 h after contact between cells and (iii) degradation of the inner cell; the inner cell degradation starts about 2 h after internalization and the cell degradation takes almost 10 h to be completed. Scale bar, 28 µm. **B** Analysis of entosis by correlative light-scanning electron microscopy. Entotic cells characterized by the nucleus (H2B-GFP, green) deformation and high concentration of actin (LifeAct-mRuby, red) in the inner cell were chosen using immunofluorescence microscopy (scale bar, 100 µm) and then the same event was analyzed by scanning electron microscopy. Entotic cells were taken with two magnifications 1000x (scale bar, 5 µm) and 2000x (scale bar, 10 µm). **C** Two populations of Hep3B cells were mixed, one population transduced with H2B-GFP, and another one transduced with H2B-RFP and transfected with siRNA targeting Rnd3. 48 h after mixing, the quantification of entotic cells was performed by evaluating the percentage of different combinations, Green in Green (wild-type in wild-type cells), Green in Red (Wild-type in siRnd3-transfected cells), Red in Green (siRnd3-transfected in wild-type) and Red in Red (siRnd3-transfected in siRnd3-transfected cells). Error bars: SD of three or more independent experiments. Significance was determined with the Mann–Whitney *U*-test. Examples of events counted after mixing of Rnd3-silencing RFP cells and wild-type GFP cells; G in G = Green in Green, G in R = Green in Red, R in G = Red in Green, R in R = Red in Red. Scale bar, 15 µm.

To investigate the CIC events induced by the loss of Rnd3, Hep3B cells expressing GFP-H2B and LifeAct-mRuby were treated with siRnd3 #1 and analyzed by time-lapse microscopy (Video 1). Entosis was initiated by the contact between the two cells, and then the internalization was marked by a high concentration of actin at the border between the two cells. The nucleus of the outer cell acquired a change in its shape due to the nucleus of the inner cell that pushes and deforms it. The final stage was characterized by the degradation of the inner cell, visible by the disappearance of the inner cell nuclei (Fig. 3A). Using correlative light-scanning electron microscopy (CLEM) allowing analysis of the same entotic cell by fluorescence microscopy and scanning EM (Fig. 3B), we found that the internalized cell has a rounded shape and seemed to be denser than the outer cell, with apparent cytoskeletal filaments. The inner cell appeared to be inside a large vacuole, the so-called entotic vacuole. The nucleus of the host cell acquired a crescent-like shape and was pushed to the cell periphery. The analysis of CLEM demonstrated that CIC structures mediated by the loss of Rnd3 are similar to entosis, showing a complete internalization of one cell inside another.

To further examine whether the loss of Rnd3 was required in inner cells, outer cells, or both, we generated Hep3B cells with green and red nuclei by expression of GFP-H2B (G) and mCherry-H2B (R), respectively (Supplementary Fig. 3A). Hep3B-mcherry-H2B were then transfected with siRnd3#1 and co-cultured with untreated Hep3B-GFP-H2B (Fig. 3C; Supplementary Fig. 3A, B). 48 h after mixing, entotic cells were analyzed by fluorescence microscopy and counted for each combination (G in G, G in R, R in G, or R in R) (Fig. 3C). Among all entotic cells, the percentage of events between wild-type cells (G in G) is about 10%, and it reaches 50% between those transfected with siRnd3#1 (R in R). This result demonstrated that the decrease of Rnd3 expression is important in both the inner and outer cells for internalization. We noted that the percentage of the combination R in G is higher than that G in R combination suggesting that the loss of Rnd3 in the inner cell could be sufficient to induce its internalization in the wild-type outer cell.

### Entosis mediated by the loss of Rnd3 is dependent on an active Rho/ROCK pathway and a decrease of E-cadherin expression

**Fig. 4:**
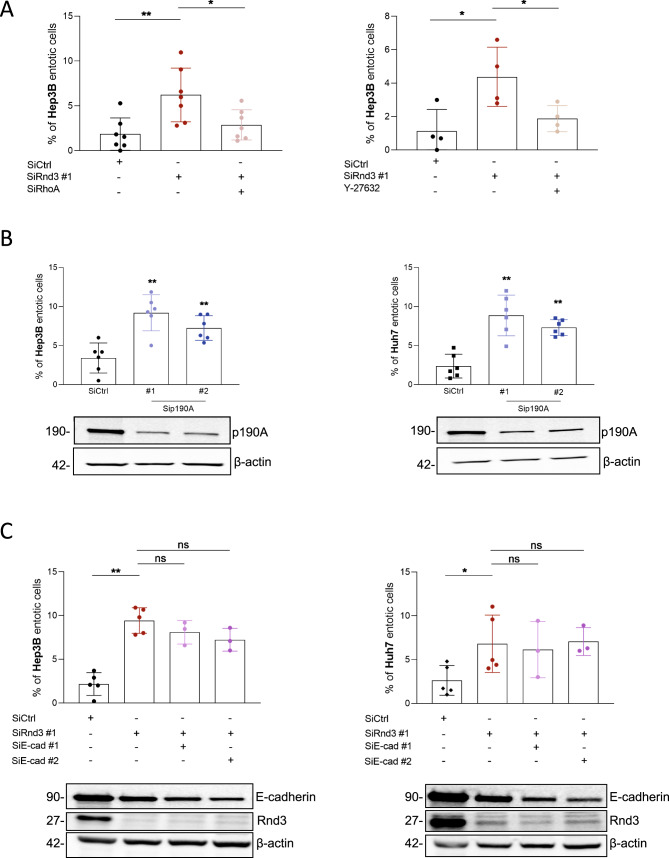
Entosis induced by Rnd3 silencing is dependent on Rho/ROCK pathway and occurs independently of E-cadherin expression. **A** Hep3B cells were transfected with siRNA targeting Rnd3 (siRnd3 #1) in the presence or absence of siRNA targeting RhoA (siRhoA) (left panel), or after treatment or not with Y-27632 (right panel) and the percentage of entosis was evaluated. **B** Hep3B or Huh7 cell lines were treated with siRNA targeting p190RhoGAP-A (siP190A #1 and siP190A #2). The Western blots show the inhibition of p190A and the graphs indicate the percentage of entotic cells. **C** Hep3B or Huh7 cells were transfected with siRNA targeting Rnd3 in the presence or absence of siRNA targeting E-cadherin (siEcad #1 or siEcad #2). The inhibition of Rnd3 and E-cadherin was confirmed by Western blot and the percentage of entotic cells was evaluated. Error bars: SD of three or more independent experiments. Significance was determined with the Mann–Whitney *U-*test.

The Rho/ROCK pathway was implicated in the entosis induced after matrix detachment and starvation. As Rnd3 is a known antagonist of this signaling pathway, we analyzed whether the RhoA/ROCK pathway was required for cell engulfment after Rnd3 silencing. We first combined RhoA and Rnd3 knockdowns in HCC cells to assess RhoA involvement (Supplementary Fig. 4A). We demonstrated that the inhibition of RhoA reverses the effect of Rnd3 silencing by decreasing the percentage of entosis in Hep3B cells (Fig. 4A, left panel). We next used the Y-27632 compound to inhibit RhoA downstream effector, ROCK. We confirmed that Y-27632 treatment was able to inhibit MYPT1 phosphorylation induced upon Rnd3 KD in Hep3B cells (Supplementary Fig. 4B). Moreover, similar to RhoA KD, we found that this treatment decreases the percentage of entosis induced by the silencing of Rnd3 (Fig. 4A, right panel). Like Rnd3, p190RhoGAP (p190A) is a negative regulator of the Rho/ROCK pathway. HCC cells were transfected with siRNA targeting p190A (Supplementary Fig. 4C). Consistently the silencing of p190A increased the percentage of entotic cells in Hep3B and Huh7 cell lines, similar results to those obtained after Rnd3 inhibition (Fig. 4B). The inhibition of p190A expression in Hep3B and Huh7 cells in the presence of siRNA targeting Rnd3 did not modify the percentage of entotic cells compared to the condition where Rnd3 expression was inhibited alone, confirming that Rnd3 and p190A act in the same pathway for the induction of entosis (Supplementary Fig. 5). Altogether, these data demonstrate that entosis mediated by the silencing of Rnd3 is dependent on the RhoA/ROCK pathway in HCC cells. Therefore, deregulation of the Rho/ROCK pathway alters the ability of HCC cells to perform entosis.

Entosis was described to be dependent on E-cadherin-based cell-cell junctions [14]. However, we have previously found a decrease in E-cadherin expression upon silencing of Rnd3 in HCC cells [17], which raises the question of the involvement of E-cadherin in the entosis observed here. We thus used E-cadherin-targeting siRNAs and analyzed their impact on entosis induced by the silencing of Rnd3 in HCC cells (Fig. 4C; Supplementary Fig. 6A). As previously published, E-cadherin expression decreased upon Rnd3 silencing in both cell types (Fig. 4C). Our results showed that inhibition of E-cadherin in Hep3B and Huh7 cells did not alter the percentage of entotic cells compared to Rnd3 knockdown alone (Fig. 4C), demonstrating that entosis mediated by Rnd3 loss is independent on E-cadherin in HCC cells. These data prompted us to explore the impact of E-cadherin silencing alone on entosis in HCC cells. Surprisingly, in contrast to breast cancer cells [21], we found that the decrease of E-cadherin expression promoted entosis in HCC cells (Supplementary Fig. 6B). We then asked whether overexpression of E-cadherin could rescue the suppression effects of Rnd3 on entosis. To do so, we overexpressed a GFP tagged-version of E-Cadherin in Rnd3-silenced Hep3B cells (Supplementary Fig. 7A). Whereas overexpression of GFP alone did not alter Rnd3 KD-induced entosis, we observed that overexpression of E-Cadherin-GFP significantly decreased the percentage of entotic cells suggesting that both Rnd3 and E-cadherin down-expression are required to promote entosis in Hep3B cells (Supplementary Fig. 7B).

### Identification of LAMP1 as an effector of Rnd3 loss-mediated entosis

**Fig. 5:**
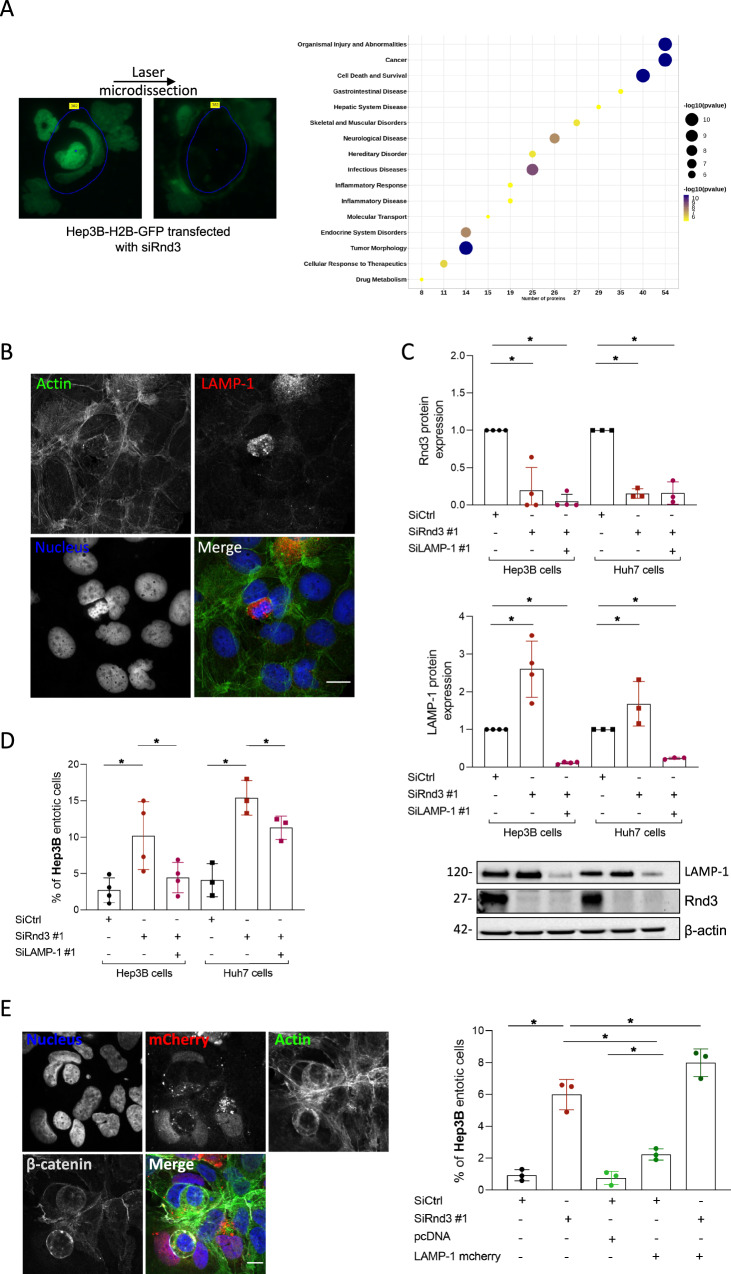
Proteomic analysis and identification of LAMP1 as an effector of Rnd3 loss-mediated entosis. **A** Hep3B-H2B-GFP cells were labeled with SILAC and transfected with siRNA targeting Rnd3 (siRnd3 #1) and the entotic cells were microdissected and analyzed using mass spectrometry. Upregulated proteins were analyzed using ingenuity pathway analysis (IPA) and represented in a bubble plot showing pathways implicated. The bubble size represents the number of upregulated proteins involved in a specific pathway. **B** Hep3B entotic cells stained to visualize nuclei (Blue), actin (Green), and LAMP1 (Red). Scale bar, 15 µm. **C** Hep3B or Huh7 cells were transfected with siRNA targeting Rnd3 in the presence or absence of siRNA targeting LAMP1 (siLAMP1 #1). The inhibition was confirmed by Western blot. The graphs show the quantification of Rnd3 and LAMP1 knock-downs in Huh7 and Hep3B cells. **D** Evaluation of entotic cells in Hep3B and Huh7 cell lines after Rnd3 and LAMP1 silencing. **E** Overexpression of LAMP1-mcherry in Hep3B cells after silencing of Rnd3. pcDNA is the control vector. Representative images of the overexpression showed by the mcherry staining and the quantification of entotic cells are shown in the right panel of (**E**). Scale bar, 15 µm. Error bars: SD of three or more independent experiments. Significance was determined with the Mann-Whitney *U*-test.

In order to better characterize the entosis mediated by Rnd3 loss, we sought to apply a global approach. However, isolation of entotic cells is not an easy task, and we failed to do so using flow cytometry. We, therefore, applied a pipeline combining isolation of entotic cells by laser microdissection in a Hep3B-H2B-GFP cell population transfected with siRnd3 #1 (Fig. 5A, left panel) and mass spectrometry-based proteomic analysis according to our published protocol [22]. We obtained 139 proteins that were underrepresented in the entotic proteome with an entosis/total proteome abundance ratio ≤0.5 and 55 enriched proteins with an entosis/total proteome abundance ratio ≥2 (Supplemental Tables 1 and 2). We used Gene Ontology (GO) to classify the identified proteins (Supplemental Tables 3 and 4). We highlighted that the underrepresented proteins were mainly involved in cellular metabolic processes with 63% (*n* = 88) of proteins associated with these biological processes. Proteins associated with intracellular anatomical structures and organelles were also found significantly decreased in entotic samples, suggesting a disassembly of cellular elements. Notably, we found a decrease in the abundance of Lamin-B1 and Lamin-B2, proteins of the nuclear envelope (Supplemental Table 2). In parallel, we found an enrichment of RNA binding proteins (42%, 23 proteins) including ribosomal and/or translation proteins (Supplemental Table 3). Network analysis using the Ingenuity Pathways Analysis platform (IPA, Qiagen) of the upregulated proteins further showed an implication of these candidates mainly in cell death/survival (Fig. 5A, right panel). We indeed found an overrepresentation of ER-associated proteins involved in stress response and/or folding of proteins such as Prdx2, HSPA5 (Bip), or protein disulfide-isomerases (P4HB, PDIA6), and PPIA, suggesting the recognition of cellular intrusion as a stressful cellular event (Supplemental Table 1). Network analysis using IPA also revealed a significantly altered functional network linked to the LAMP1 protein in entotic cells, where proteasome, chaperone, and ER stress proteins were also present (Supplemental Fig. 8A). LAMP1 is known to be implicated in the degradation of the inner cells by autophagy during the entotic mechanism. We confirmed the high expression of LAMP1 in the inner cells in the early stage of degradation (Fig. 5B). To validate the involvement of LAMP1 in entosis induced by Rnd3 silencing, we inhibited Rnd3 in the presence or absence of siRNA targeting LAMP1 in Hep3B and Huh7 cells (Fig. 5C). Whereas, as previously shown, the silencing of Rnd3 increased the percentage of entosis in both cell lines, this percentage returned to control levels when Rnd3 and LAMP1 were co-silenced (Fig. 5D). These data demonstrated the involvement of LAMP1 not only in the final stage of inner cell degradation but also in the overall mechanism of entosis induced by silencing of Rnd3. Interestingly, using RNAseq analysis of Hep3B cells (data not shown), *LAMP1* gene expression was found upregulated upon Rnd3 silencing (Supplemental Fig. 8B). These data were confirmed at the protein level in both Hep3B and Huh7 cells (Fig. 5C, bottom panel). This up-regulation of LAMP1 upon Rnd3 silencing appears to be dependent on RhoA as RhoA-KD rescued LAMP1 protein level (Supplemental Fig. 8C), suggesting that LAMP1 and RhoA pathway are related factors. We further analyzed whether the overexpression of LAMP1 is sufficient to promote entosis. The ectopic expression of LAMP1 in Hep3B cells only slightly increased the percentage of entotic cells, which remains very low compared to that obtained with Rnd3-silencing alone. However, the combination of overexpression of LAMP1 and Rnd3 inhibition significantly increased the entosis percentage compared to Rnd3-silencing alone (Fig. 5E). All these results suggest that Rnd3 favors entosis through LAMP1 expression and function. Thus, LAMP1 is an important factor to be considered all over the entotic mechanism.

### Entosis in patient tumors correlates with invasive features

**Fig. 6:**
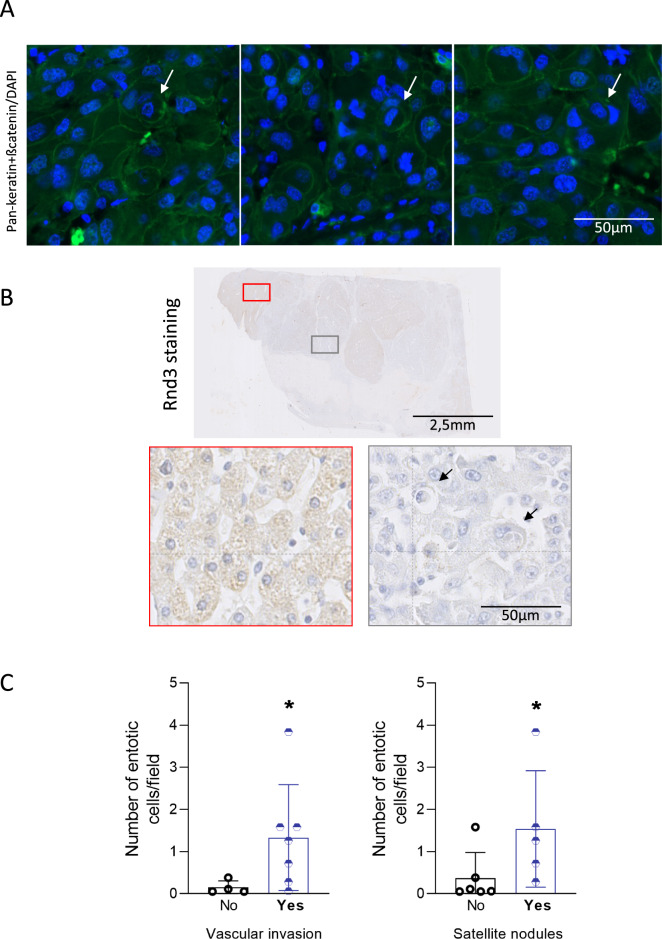
Entosis in patient tumors correlates with invasive features. **A** Representative entotic cells in HCC tumor tissues stained with DAPI (Blue) and pan-keratin/bate-catenin (Green). Scale bar, 50 µm. **B** HCC tissues stained using hematoxylin staining, and Rnd3 antibody. Scale bar: 2.5 mm. Entotic cells were evaluated in the positive (red) or negative (gray) Rnd3 areas. Scale bar: 50 µm. **C** Correlation between the number of entotic cells and the presence (Yes) or not (No) of satellite nodules or vascular invasion. *N* = 10 patient samples, significance was determined with the Mann–Whitney *U-*test.

We next investigated the presence of entotic cells in HCC patient tissues. Using membrane (pan-keratin/β-catenin) and nucleus staining, we found few entotic cells in tumor samples (Fig. 6A). These events were rare, but visible in the tumor part, using an immunofluorescence approach on tissues. We then performed Rnd3 staining on HCC sections and looked for entotic cells in negative and positive Rnd3 areas. We noticed that the entotic cells are present in tumor sections with low expression of Rnd3 (Fig. 6B) supporting our in vitro data concerning the implication of Rnd3 downregulation in the entosis process. Using a cohort of 10 patient samples (Table 1), we then correlated the number of entotic cells with the characteristics of patient tumors. Similar to the low level of Rnd3 expression [17], we found that the number of entotic cells was significantly higher in tumors with satellite nodules or vascular invasion, which are indicative of local invasion of HCC (Fig. 6C). All these results suggest the association between the entotic events, loss of Rnd3 and tumor progression.

**Table 1:** Clinical and tumoral characteristics of patients.

Clinical characteristics	Value
Age (mean ± standard deviation)	69.5 ± 9.1 years
Gender (male)	100%
HBV infection	0%
HCV infection	44.4%
Cirrhosis	44.4%
**Tumor characteristics**	**Value**
Diameter (mean ± standard deviation)	56.63 ± 39.76
Satellite nodule	50%
Vascular invasion	70%

## Discussion

In this study, we characterized entosis in liver cancer cells, which resembles to that largely described in the literature in breast cancer cells [23]. We demonstrated that entosis could be induced by matrix detachment and nutrient deprivation in HCC cells. In addition, we identified that entosis can be efficiently triggered in HCC cells through the loss of *RND3* expression. Others and we previously described the downregulation of this gene expression in human HCC [17, 18, 24]. Moreover, Rnd3 loss was associated with liver cancer cell proliferation, invasion, chemoresistance, and senescence [17–19, 25]. We revealed here that Rnd3 loss also favors entosis in HCC tumors. Recently, Rnd3 has been involved in the p53-dependent entotic mechanism driven by transient mitotic arrest in breast cancer cells [26]. However, in contrast to our results, it was found that Rnd3 expression under the regulation of p53 was essential to promote entosis in breast cancer cells. Thus, the Rnd3 role appears to be different in breast and liver cancers. In this line, Rnd3 expression does not seem to be regulated by p53 in HCC cells as no correlation between *RND3* expression and TP53 mutations could be established in HCC human samples [17].

Rnd3 is a negative regulator of the RhoA/ROCK pathway. Herein, we confirmed the strong dependency of the entotic mechanism on this pathway. We found that entosis induced by the loss of *RND3* is reduced after the inhibition of the Rho/ROCK pathway. Mechanically, entosis depends on actomyosin contractility regulated by the RhoA GTPase activity in the invading cell. It was described that outer cells have lower actomyosin contractility, consistent with a more deformable status compared to invading cells. In our co-culture experiments, the percentage of wild-type cells inside Rnd3-negative cells was very low, suggesting that the penetrating cells require an increase in contractility. However, in liver cancer cells, the loss of Rnd3 is important in both the inner and outer cells, as a maximum of entosis was found when Rnd3 expression is decreased in both the inner and the outer cells. This result is consistent with the existence of a required threshold for Rnd3 expression to favor entosis. Thus, a knockdown recreating an imbalance in contractility, in agreement with the literature, the more rigid cell could be internalized by a more deformable cell [21]. Consistently, we observed a high concentration of actin and the presence of fibers in the inner cells by electronic microscopy.

Several studies have shown the importance of E-cadherin in entosis induced by matrix detachment and starvation [10, 11, 21]. Interestingly, we showed that entosis induced by the silencing of Rnd3 occurs independently of E-cadherin, and is even altered upon E-Cadherin re-expression. Consistent with our published results showing the decrease of E-cadherin expression upon silencing of Rnd3 in HCC cells [17], we even found that loss of E-cadherin favors entosis in Hep3B and Huh7 cells. Thus, entosis induced by the loss of Rnd3 could be dependent on other transmembrane proteins that may trigger cell-cell interaction.

A proteomic analysis of laser-captured entotic cells allowed us to identify an overrepresentation of LAMP1 and ER-associated proteins involved in stress response and/or folding of proteins. Statistical analyses by Gene Set Enrichment Analysis did not reveal significant enrichment of canonical pathways of cellular degradation or stress. However, a functional environment of the LAMP1 protein is significantly modified with key proteins of these pathways. It is possible that entosis pathways involve only certain proteins known to be implicated in degradation and stress and mobilize a specific protein set. Our data confirm that entotic cells express highly LAMP1, a protein described in the literature as implicated in the final stage of entosis, i.e., degradation of inner cells. Indeed, the membrane of the entotic vacuole surrounding the internalized cells recruits LAMP1 and LC3 independently of the autophagosome formation. The fusion between the entotic vacuole and the lysosome of outer cells allows to degrade the inner cells [27]. Herein, we highlighted that *LAMP1* expression is upregulated at the mRNA and protein levels upon Rnd3 silencing, in a RhoA-dependent manner. Our results are consistent with an involvement of LAMP1 in inner cell degradation after internalization induced by the loss of Rnd3. However, we found that LAMP1 is involved not only in degradation but also in the whole mechanism of entosis. LAMP1 could be among the different signals necessary for internalization. Although LAMP1 primarily resides at the lysosomal membrane, its localization to cell surface expression was described to mediate cell-cell adhesion and favor melanoma cell invasion [28].

The degradation of entotic cells could serve to feed cells, supporting the survival of the outer tumor cells in stress conditions. Entosis may also contribute to tumorigenesis by inducing aneuploidy [29]. Others and we previously described Rnd3 as a potential metastasis suppressor as its down-expression was associated with poor prognosis in HCC patients. The results presented here support the hypothesis that loss of Rnd3 could participate in HCC progression through the promotion of entosis. Accordingly, entosis in HCC patient tissues correlates with the presence of satellite nodules and vascular invasion. Thus, targeting the entotic mechanism may be valuable as a novel therapeutic avenue to impair HCC progression.

## Materials/subjects and methods

### Cell culture

The liver cancer cell lines (Hep3B, Huh7, HepG2, Huh6) and the breast adenocarcinoma cell line MCF-7 were cultured in Dulbecco modified Eagle’s medium (DMEM 1x glutamax, Fisher Scientific) supplemented with 10% heat-inactivated fetal bovine serum and incubated at 37 °C in a humidified 5% CO2 atmosphere. Cell line authentication was performed using short tandem repeat analysis, and the absence of mycoplasma contamination in cell culture media was tested every week. The nutrient deprivation was performed by culturing the cells in DMEM 1x glutamax with a very low concentration of glucose (Fisher Scientific) and supplemented with 10% of dialyzed heat-inactivated FBS for 10–13 h. For induction of cell suspension, the adherent cancer cells were trypsinized and cultured in non-treated plastic Petri dishes for 13 h. Stable cell lines with fluorescent nuclei or fluorescent F-actin were generated through transduction with lentiviruses expressing H2B-GFP (Addgene #25999), H2B-RFP (Addgene #26001) or LifeAct-mRuby. Transient knockdowns were done by transfection of small interfering RNAs (siRNAs) into cells using the lipofectamine RNAi max (Invitrogen) according to its manufacturer’s protocol. siRNAs targeting Rnd3 (SiRnd3 #1, SiRnd3 #2, SiRnd3 #3), p190RhoGAP-A (Sip190RhoGAP-A #1, Sip190RhoGAP-A #2), RhoA and E-cadherin (siE-cadherin #1) were purchased from Eurofins Genomics and the sequences are presented in Supplemental Table 5. siE-cadherin #2 were purchased from Thermo Fisher Scientific (CDH1, cat#: 4427037, ID: s2768). Control siRNA corresponds to AllStars Negative control from Qiagen. Transient transfection of plasmids was performed using Lipofectamine^TM^ 3000 reagent according to the manufacturer’s protocol (Thermo Fisher Scientific). The E-Cadherin-GFP construct was a generous gift from Dr. Peter Coopman (Montpellier). To induce a stable suppression of endogenous Rnd3 expression, we used Hep3B-shRnd3 and Huh7-shRnd3 cell lines with conditional, doxycycline-dependent, expression of shRnd3 as described previously [30]. Hep3B-shCtrl and Huh7-shCtrl cell lines, conditionally expressing the control shRNA targeting the firefly luciferase, were used as controls. Stable cell lines were cultured as described above and shRNA expressions were induced by 50 mg/mL of doxycycline. For ROCK inhibition, cells were treated with Y-27632 (Sigma Aldrich) at 5 µM for 24 h. Sorafenib (Bay 43–9006, Enzo Life Science) was used at two doses 8 or 10 µm to treat Huh7 cell lines.

### Antibodies, immunoblot analysis

Cells were lysed in RIPA lysis Buffer (Sigma) supplemented with protease inhibitor cocktail (Roche Diagnostics) and protein concentration was determined using Bio-Rad Protein Assay (Lowry). 60 μg of proteins from each sample were separated on 10% polyacrylamide gel (Bio-Rad) and blotted onto nitrocellulose membranes (0.2 μm nitrocellulose, Bio-Rad) using Trans-Blot Turbo Transfer System (Bio-Rad). The membranes were blocked in Odyssey blocking buffer for 30 min and then incubated with each of the following specific primary antibodies: Mouse anti-RhoE/Rnd3 (1:1000, clone 4, Cell Signaling, #3664 S), Rabbit anti-actin (1:2000, Sigma Aldrich, #A2066), Mouse anti-HSP90 (1:1000, Santa Cruz, #sc-69703), Rabbit anti-Mypt-1 (1:500, Millipore, #07-672-I), Rabbit anti-phospho-Mypt-1 (Thr696) (1:500, Millipore, #ABS45), Mouse anti-RhoA (1:1000, Santa Cruz, #sc-418), Mouse anti-p190A (1:1000, BD Biosciences, #610149), Mouse anti-E-cadherin (1:1000; BD Biosciences, #610182), Mouse anti-HSP90 (1:1000, Santa Cruz, #sc-69703), Mouse anti-LAMP1 (1:1000, Santa Cruz, #sc-20011), Rabbit anti-GFP (1/1000, Abcam, #ab290) either 1 h at room temperature or overnight at 4 °C. After incubation with the specific secondary antibodies, all blots were analyzed with the Bio-Rad Chemidoc system.

### Immunofluorescence staining

The following primary antibodies were used for immunofluorescence (IF): Mouse anti-beta-catenin (1:400; BD Biosciences, #610154), Mouse anti-E-cadherin (1:50; BD Biosciences, #610182), Mouse anti-LAMP1 (1:100, Santa Cruz, #sc-20011). IF was performed on adherent or suspended cells after cytospin using the Shandon Cytospin 2 centrifuge at 110 rpm for 5 min. Cells were fixed and permeabilized in 4% paraformaldehyde PFA (Electron Microscopy Science, #15710) and 1% Triton X-100 respectively for 10 min at RT, followed by PBS washing and blocking in PBS-5%BSA for 20 min. Cells were then incubated with primary antibodies for 45 min followed, after PBS washing, by a incubation with the secondary antibodies (Interchim) specific for primary antibodies. F-actin was stained using fluorescent phalloidin (Molecular Probes). Finally, the cells were counterstained with DAPI (Sigma, #D9542) and the coverslip mounting on the glass slide was done using Fluoromount-G medium (Interchim #FP-483331).

### Time-lapse microscopy

Hep3B cells expressing H2B-GFP and Lifeact-mRuby were cultured on glass-bottom dishes 35 mm high (Ibidi) after Rnd3 silencing, and time-lapse microscopy was performed in 37 °C and 5% CO_2_ live-cell incubation chambers. The fluorescence was acquired every 2 h for 48 h using the spinning-disk LiveSR confocal microscope. The image analysis and the video reconstitution were done using ImageJ program.

### Correlative light-electron microscopy (CLEM)

Hep3B cells expressing H2B-GFP were transfected with siRNA targeting Rnd3 (SiRnd3 #1) and cultured on glass coverslips gridded and numbered (Delta microscopy, #72265-12). After selection of the area of interest containing the entotic events using fluorescent microscopy, cells were fixed with 2.5% glutaraldehyde (Electron Microscopy Science, #15960) for 30 min at RT followed by incubation at 4° for 1 h in the dark. After washing with Sorensen’s phosphate buffer 0.2 M, pH 7.4 (Electron Microscopy Science, #11601-10), cells were dehydrated in graded series of ethanol solutions, including 50%, 70%, 90% for 5 min and 100% for 2 times, 5 min at RT in the dark. Finally, the cells were dried with CO_2_ and observed using a Zeiss GeminiSEM300 microscope.

### Entosis quantification

The entotic event is defined by 1) the presence of a cell inside another one with a deformed nucleus and 2) the high concentration of actin staining around the inner cell. The entotic cells were observed with epifluorescence microscopy (Zeiss). Quantification was done by counting 300–400 of total cells for each condition. The percentage of entotic events was calculated by dividing the number of entotic events by the total.

### SILAC labeling, laser capture microdissection, and proteomic analysis

To discriminate laser-captured proteins from undesirable exogenous contaminating proteins, Hep3B-H2B-GFP cells were first metabolically labeled using stable isotope labeling with amino acids in cell culture (SILAC) method, as previously published [22]. For that, Hep3B-H2B-GFP cells were cultured in DMEM medium (Dulbecco’s modified Eagle’s medium, Invitrogen) supplemented with 10% dialyzed fetal bovine serum, 200 mg/L L-proline, and 84 mg/L l-Arginine and Lysine. The incorporation of labeled amino acids was done after six cycles of cellular doubling. After transfection with siRnd3 #1, cells were seeded for 12–24 h on LamPen coated with collagen matrix allowing cells to adhere. Cells were fixed with PFA and the laser capture microdissection was performed using PALM type 4 micro-beam (Zeiss). SILAC-labeled cells were transfected with Rnd3-targeting siRNAs and entotic cells with crescent-shaped nuclei were microdissected. To obtain enough material for proteomic analysis, we manually collected 2000 cells in triplicate. With 2000 isolated entotic cells, we have identified a mean of 2880 ^13^C peptides corresponding to 406 proteins with at least 2 specific peptides. Given the small quantity of material analyzed, the first step guarantees the specificity of identifications by distinguishing labeled proteins from microdissected cells from unlabeled environmental contaminants (keratins, skin and hair proteins, etc…). In parallel, a standard range (500 ng to 5 ng) of protein quantity was done on cells labeled with SILAC and transfected with control siRNA (siCtrl) in order to compare the same quantity of entotic cells microdissected and obtained after transfection with siRnd3 to cells transfected with siCtrl. Three independent biological replicates on total protein extracts from SILAC-labeled cells were compared by label-free protein quantification. Proteins were loaded on a 10% acrylamide SDS-PAGE gel and proteins were visualized by Colloidal Blue staining. Migration was stopped when samples had just entered the resolving gel and the unresolved region of the gel was cut into only one segment. The steps of sample preparation and protein digestion by the trypsin were performed as previously described [31]. NanoLC-MS/MS analysis was performed using an Ultimate 3000 RSLC Nano-UPHLC system (Thermo Scientific, USA) coupled to a nanospray Orbitrap Fusion™ Lumos™ Tribrid™ Mass Spectrometer (Thermo Fisher Scientific, California, USA). Each peptide extracts were loaded on a 300 µm ID x 5 mm PepMap C_18_ precolumn (Thermo Scientific, USA) at a flow rate of 10 µL/min. After a 3 min desalting step, peptides were separated on a 50 cm EasySpray column (75 µm ID, 2 µm C_18_ beads, 100 Å pore size, ES903, Thermo Fisher Scientific) with a 4–40% linear gradient of solvent B (0.1% formic acid in 80% ACN) in 57 min. The separation flow rate was set at 300 nL/min. The mass spectrometer operated in positive ion mode at a 2.0 kV needle voltage. Data was acquired using Xcalibur 4.4 software in a data-dependent mode. MS scans (*m*/*z* 375–1500) were recorded at a resolution of R = 120000 (@ *m*/*z* 200), a standard AGC target, and an injection time in automatic mode, followed by a top speed duty cycle of up to 3 s for MS/MS acquisition. Precursor ions (2 to 7 charge states) were isolated in the quadrupole with a mass window of 1.6 Th and fragmented with HCD@28% normalized collision energy. MS/MS data was acquired in the ion trap with rapid scan mode, a 20% normalized AGC target, and a maximum injection time in dynamic mode. Selected precursors were excluded for 60 s. Protein identification was done in Proteome Discoverer 2.5. Mascot 2.5 algorithm was used for protein identification in batch mode by searching against a UniProt *Homo sapiens* protein database (75796 entries, released September 3, 2020; https://www.uniprot.org/ website). Two missed enzyme cleavages were allowed for the trypsin. Mass tolerances in MS and MS/MS were set to 10 ppm and 0.6 Da. Oxidation (M), acetylation (K), SILAC modifications (K, R) were searched as dynamic modifications, and carbamidomethylation (C) as static modifications. Raw LC-MS/MS data were imported into Proline Studio for feature detection, alignment, and quantification. Protein identification was accepted only with at least 2 specific peptides with a pretty rank=1 and with a protein FDR value less than 1.0% calculated using the “decoy” option in Mascot. Label-free quantification of MS1 level by extracted ion chromatograms (XIC) was carried out with parameters indicated previously [31]. The normalization was carried out on the median of ratios. The inference of missing values was applied with 5% of the background noise. The mass spectrometry proteomics data have been deposited to the ProteomeXchange Consortium via the PRIDE partner repository [32] with the dataset identifier PXD043640.

### Immunohistochemistry on HCC samples

Mouse HCC samples were from Hep3B xenografts previously described [19]. This model consisted of subcutaneous inoculation of Hep3B-shCtrl or Hep3B-shRnd3 cells in immunodeficient NOG mice treated with doxycycline to induce or not Rnd3 knockdown. The analysis of entosis was performed on tumor sections stained with hematoxylin to mark the cytoplasm and nuclei of cells. Human samples came from resected or explanted livers of HCC patients treated in Bordeaux from 1992–2005. The characteristics of HCCs used for the IHC analysis (10 HCCs) are indicated in Table 1. Formalin-fixed, paraffin-embedded sections of human or mouse HCC samples were used for Hematoxylin-Eosin or immunohistochemical staining. Staining of Rnd3 and pan-keratin/beta-catenin was performed as previously published [17, 33].

### Statistical analysis

The frequency of entotic cells was represented as a percentage (mean ± SD) of the total counted cells for at least three experiments. Data were analyzed using GraphPad Prism 10. For all experiments, significance was determined with the Mann–Whitney *U*-test, *p < 0.05, **p < 0.01*,* ***p *<* 0.001.

### Supplementary information

Supplemental Figures

Supplemental Table 1

Supplemental Table 2

Supplemental Table 3

Supplemental Table 4

Supplemental Table 5

The video shows the entosis mechanism upon Rnd3 silencing in Hep3B cells

Supplemental legends

Original Data File

## Data Availability

The mass spectrometry proteomic data have been deposited to the ProteomeXchange consortium with the dataset identifier PXD043640. Additional data are available from the corresponding author upon reasonable request.
