# Supplementary material for: Loss of RND3/RHOE controls entosis through LAMP1 expression in hepatocellular carcinoma
Source: Cell Death Dis. 2024 Jan 13;15(1):46. doi: 10.1038/s41419-024-06420-3 (PMC10787830; doi:10.1038/s41419-024-06420-3)

Supplemental Figure 1

A

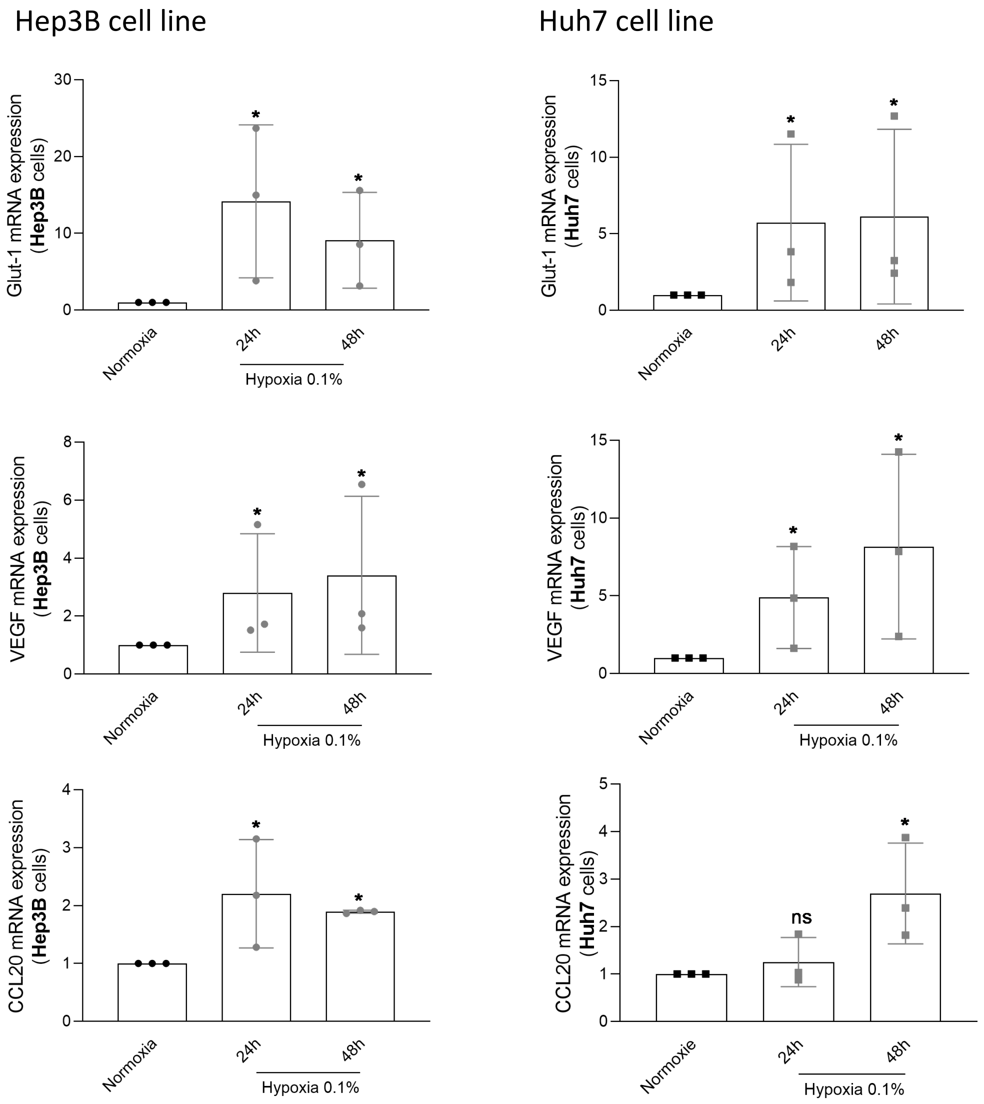

B

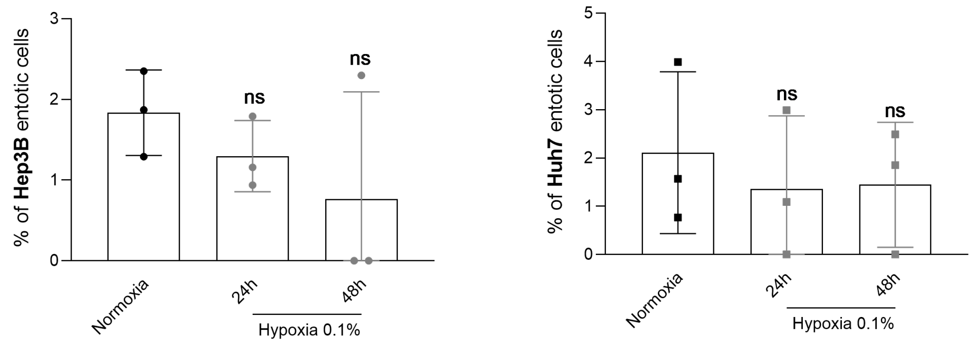

C

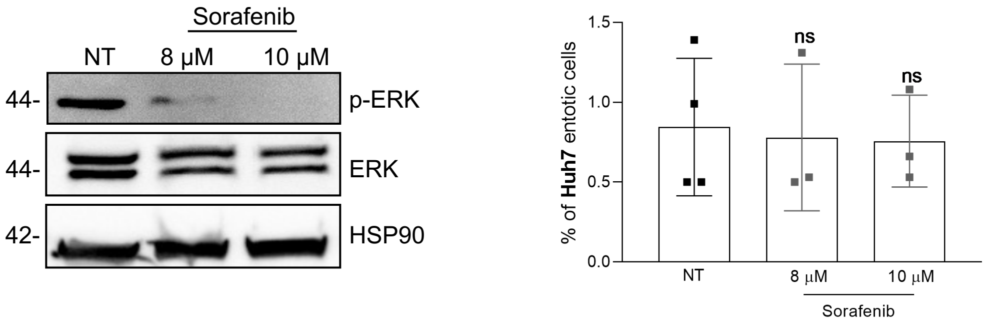

Supplemental Figure 2

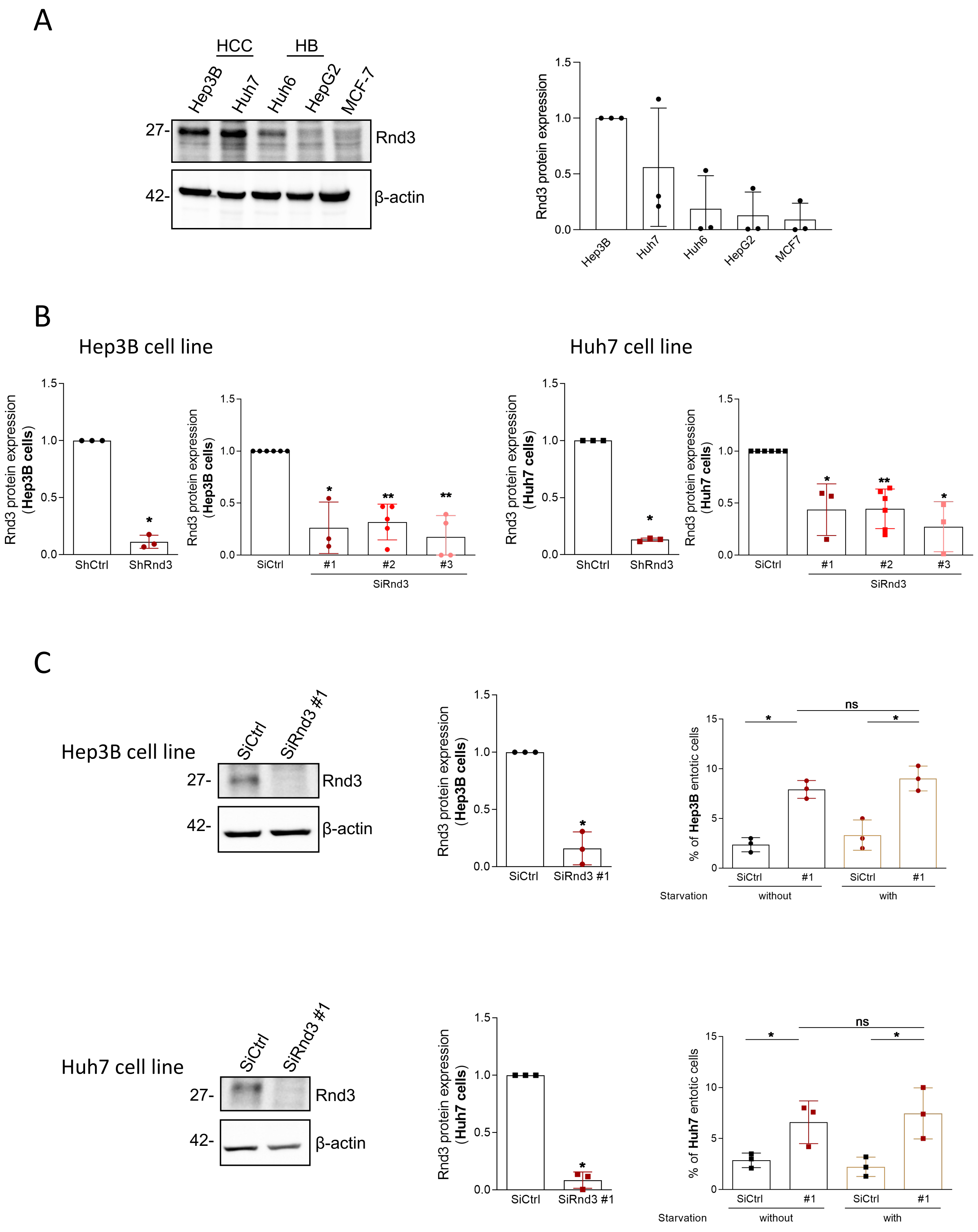

Supplemental Figure 3

A

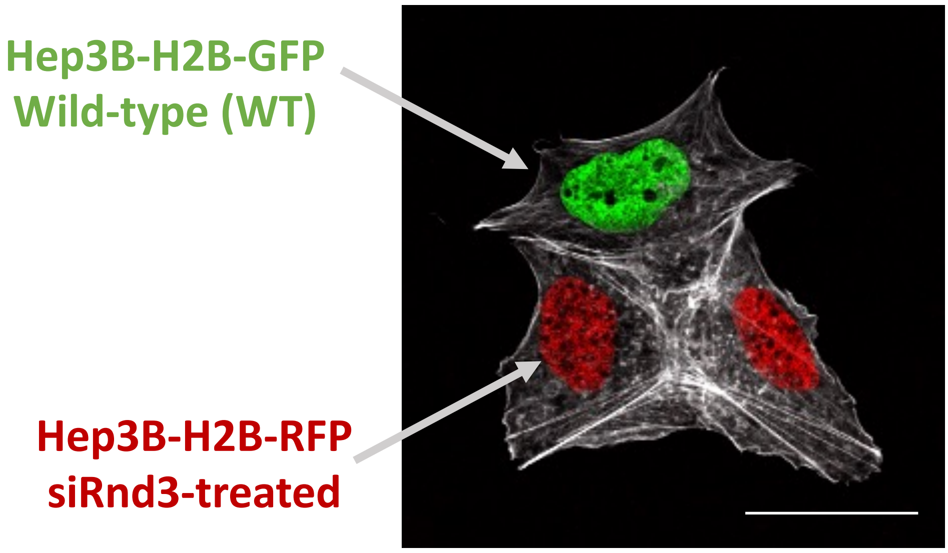

B

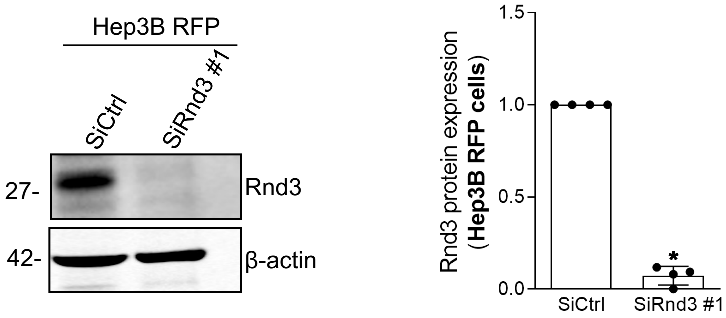

Supplemental Figure 4

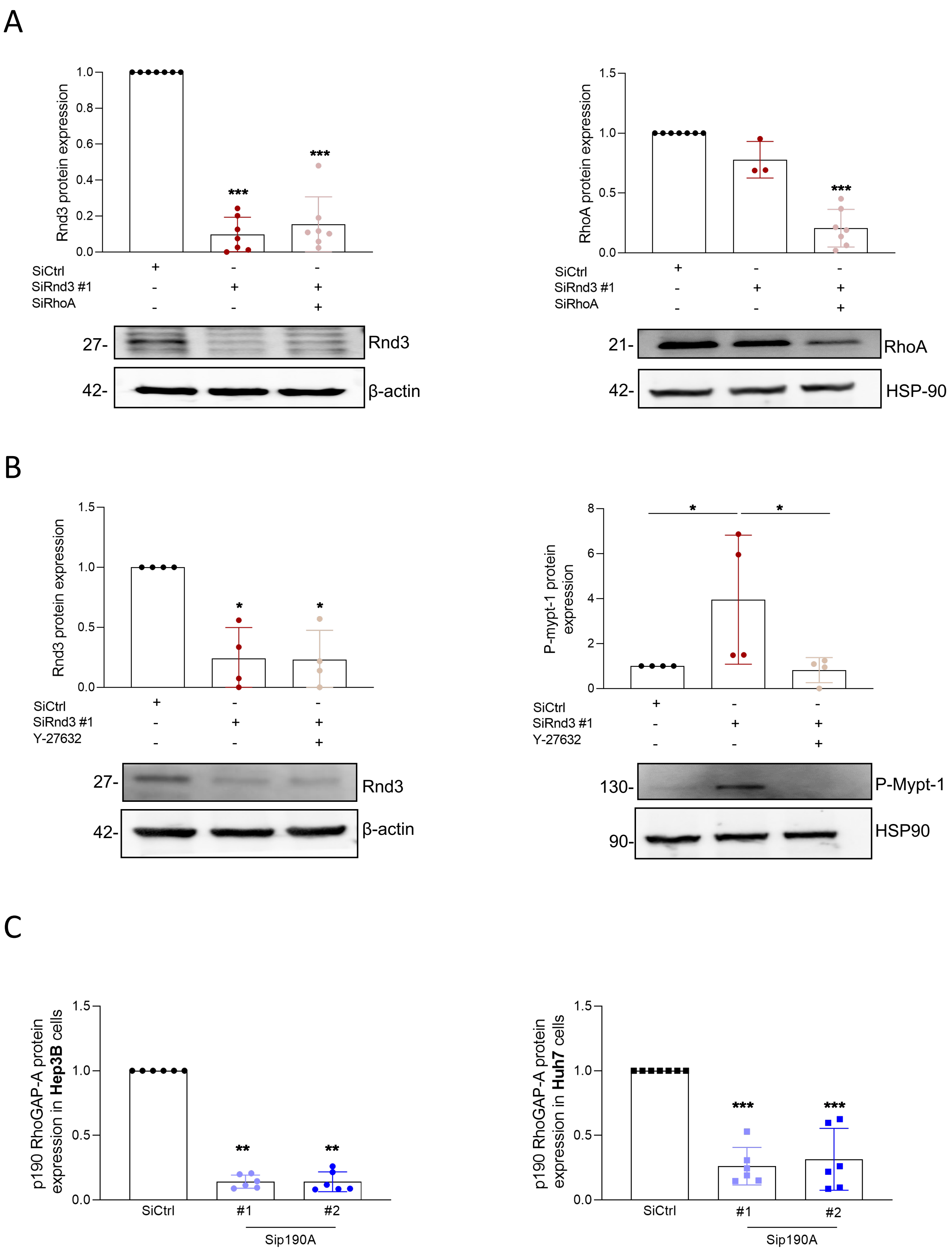

Supplemental Figure 5

A

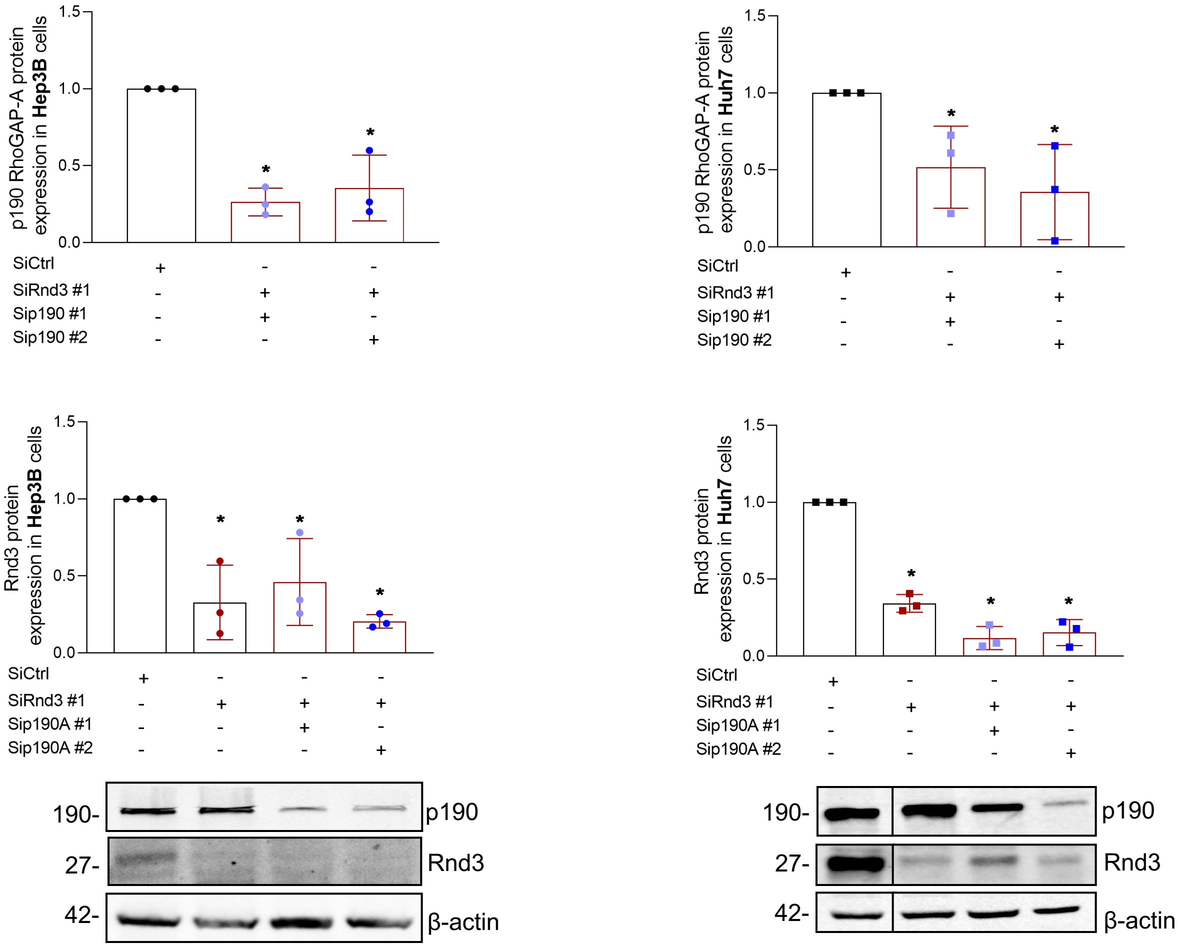

B

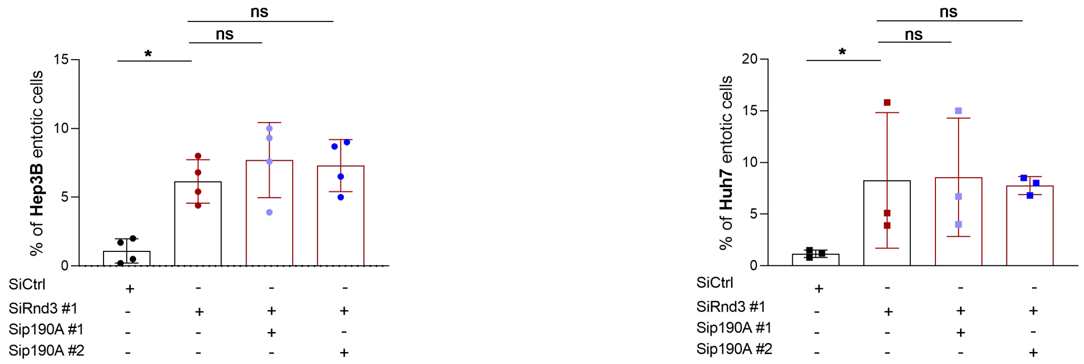

Supplemental Figure 6

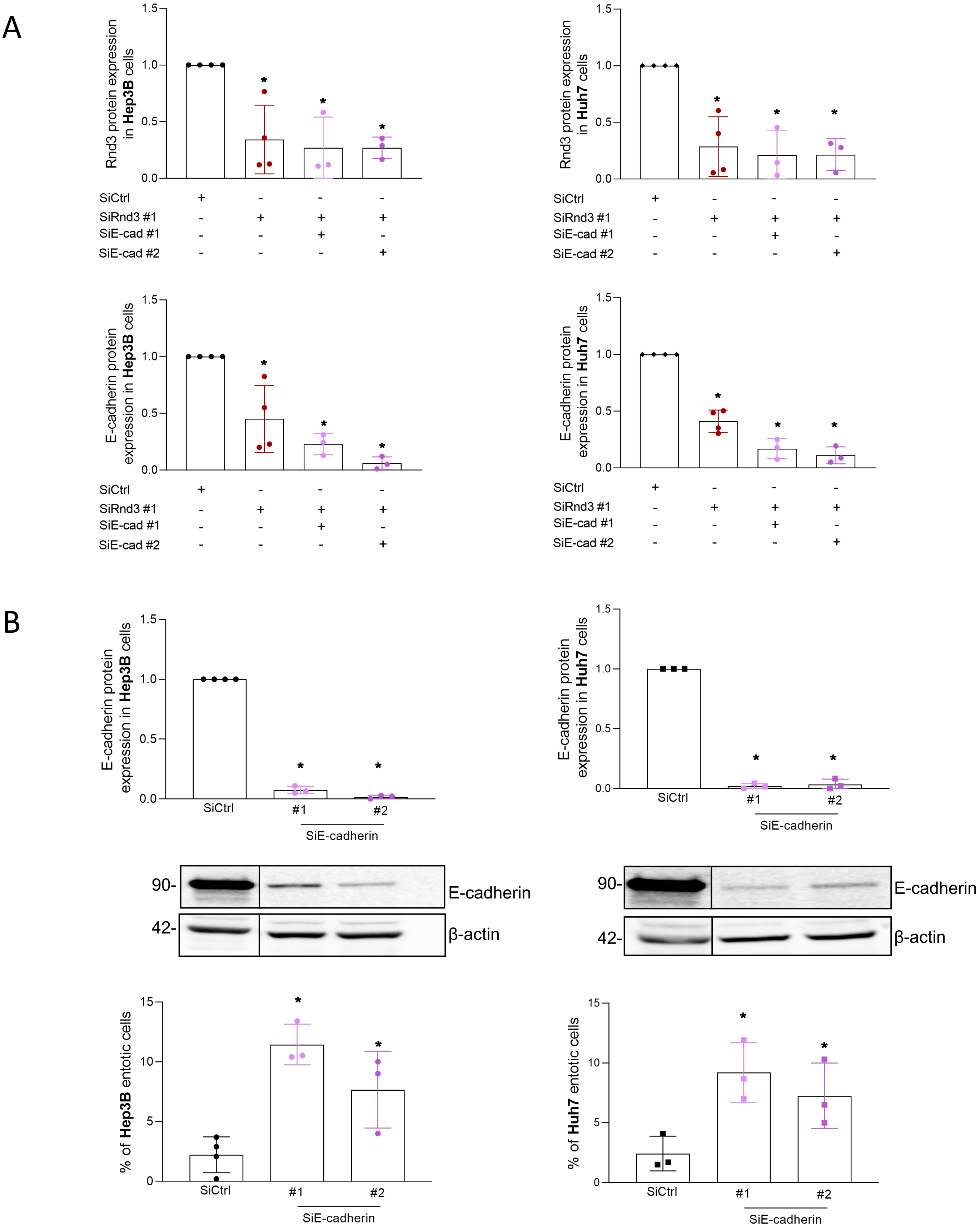

Supplemental Figure 7

A

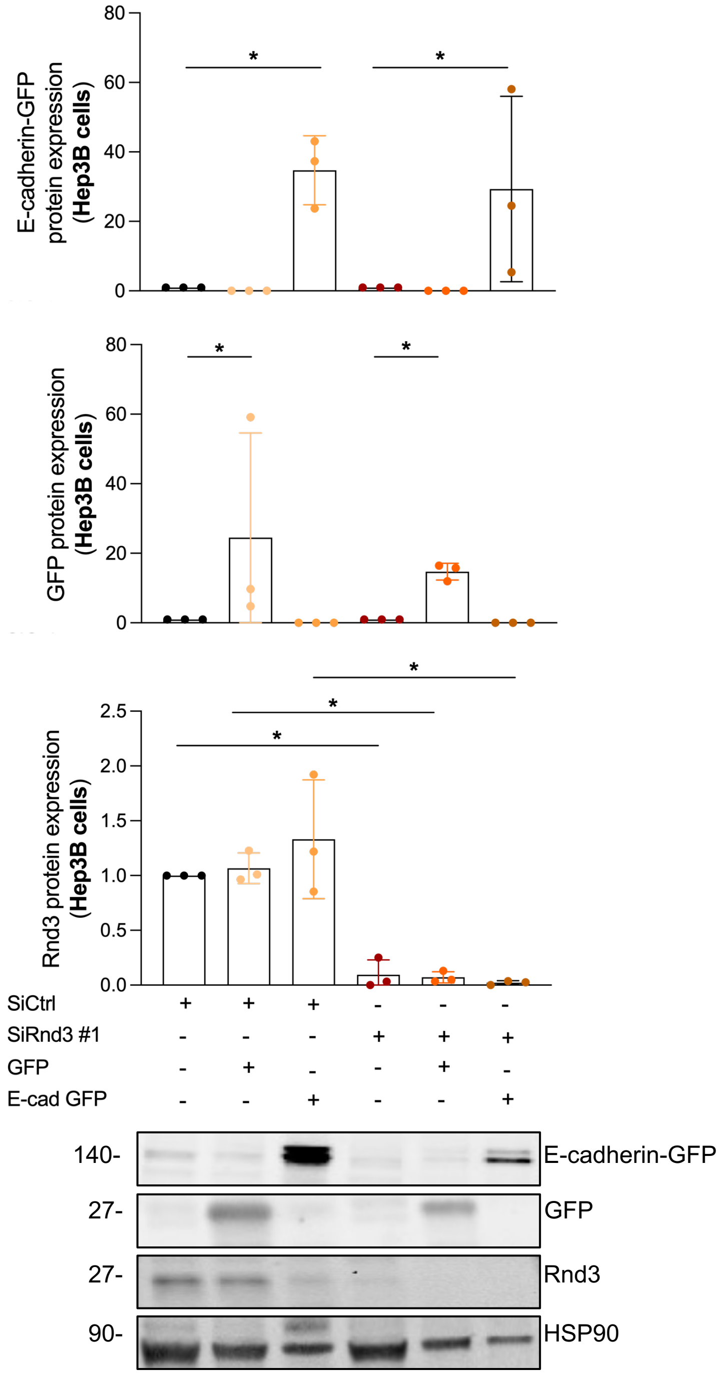

B

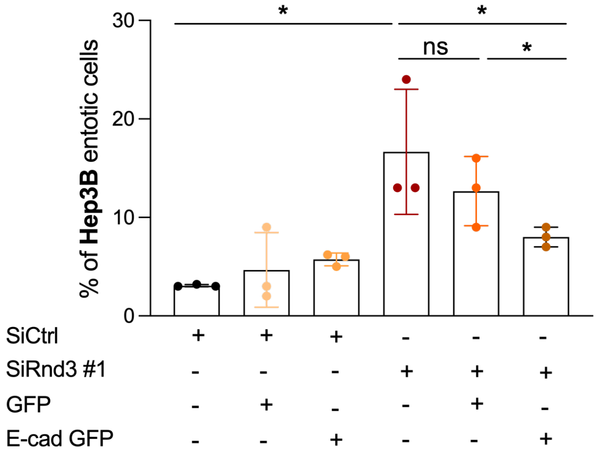

Supplemental Figure 8

A

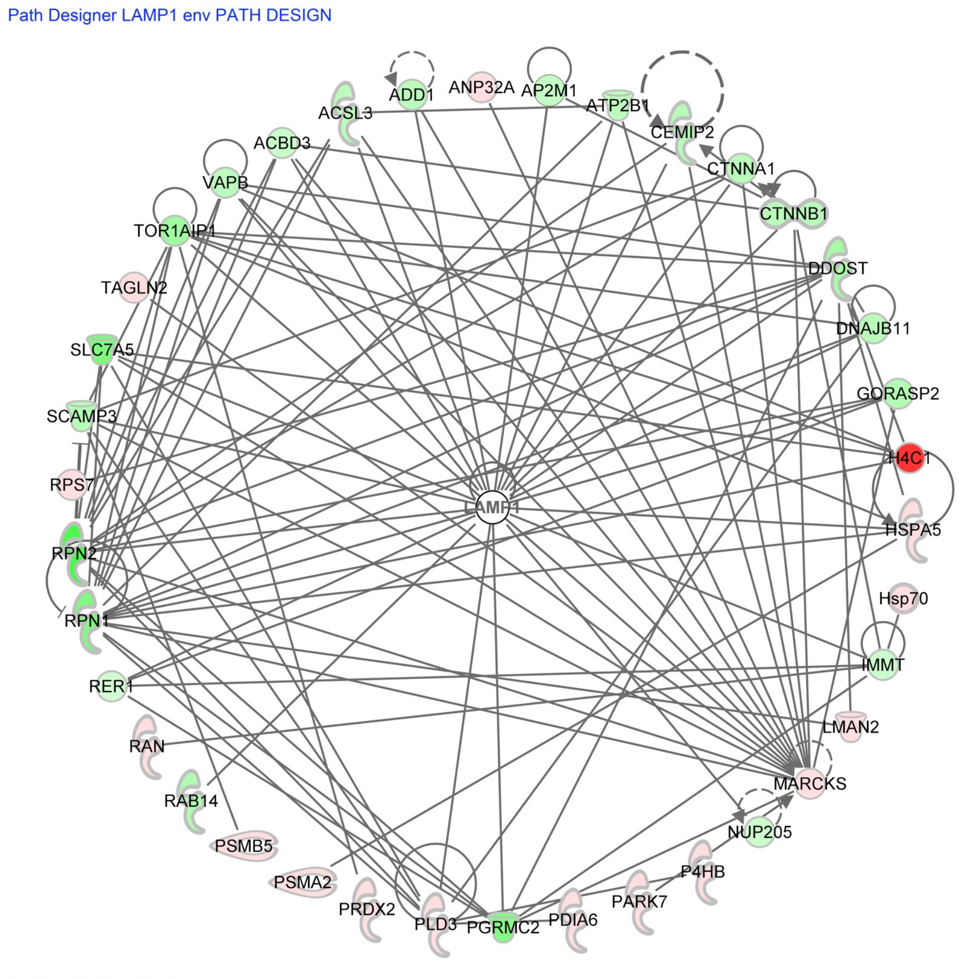

© 2000-2023 QIAGEN. All rights reserved.

B

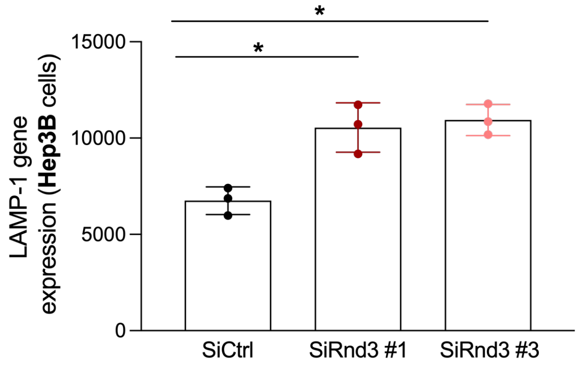

C

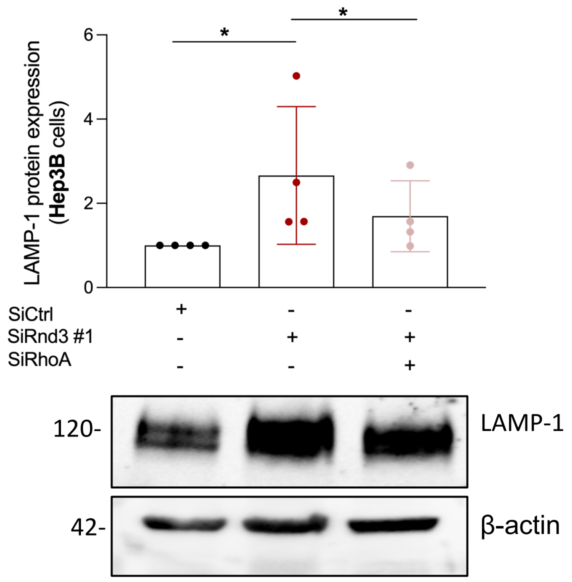

Supplement: Supplementary file 1 — Supplemental Figures [file 41419_2024_6420_MOESM1_ESM.pdf]
