# Supplementary material for: Loss of RND3/RHOE controls entosis through LAMP1 expression in hepatocellular carcinoma
Source: Cell Death Dis. 2024 Jan 13;15(1):46. doi: 10.1038/s41419-024-06420-3 (PMC10787830; doi:10.1038/s41419-024-06420-3)
Supplement: Supplementary file 8 — Supplemental legends [file 41419_2024_6420_MOESM8_ESM.docx]

*Supplemental information*

*Supplemental figure legends*

**Supplemental Figure 1: (A) Hypoxia and Sorafenib treatments do not induce entotic events in Hep3B and Huh7 cell lines.**

**(A)** Quantification of mRNA levels of GLUT1, VEGF, CCL20 in Hep3B (left panel) and Huh7 (right panel) cell lines 24h or 48h after hypoxia (0.1% O2) compared to cells grown in normoxia condition. **(B)** Evaluation of percentage of Hep3B and Huh7 entotic cells after hypoxia. **(C)** Huh7 cell line treated with two doses of Sorafenib (8, 10 µM) for 72 hours and confirmed by Western Blot showing the inhibition of p-ERK compared to the total ERK. The right panel presented the quantification of entotic events in these conditions. Error bars: SD of three independent experiments. Significance was determined with the Mann Whitney U test.

**Supplemental Figure 2: The combination of Rnd3 silencing and starvation does not increase the entosis mechanism in Hep3B and Huh7 cells compared to that induced by Rnd3 silencing alone.**

**(A)** Comparison of the Rnd3 protein expression between HCC (Hep3B, Huh7), HB (Huh6, HepG2) and MCF7 breast cancer cell lines. **(B-C)** Evaluation of entotic events in Hep3B **(B)** and Huh7 **(C)** after silencing of Rnd3 combined or not with starvation. Error bars: SD of three independent experiments. Significance was determined with the Mann Whitney U test.

**Supplemental Figure 3: Role of Rnd3 silencing in the inner or outer cells.**

**(A)** Two populations of Hep3B cells were mixed, one population transduced with H2B-GFP and another one transduced with H2B-RFP and transfected with siRNA targeting Rnd3. Image of Rnd3-silencing RFP cells and wild-type GFP cells co-culture. Scale bar, 15 µm. **(B)** Rnd3 knock-down was assessed by Western blot, β-actin is the loading control. The graph shows quantification of entotic cells upon Rnd3 KD. Error bars: SD of four independent experiments. Significance was determined with the Mann Whitney U test.

**Supplemental Figure 4: Validation of Rnd3, RhoA, p190RhoGAP-A inhibition by western blot.**

**(A-B)** Western blot and quantification of Rnd3 and RhoA knock-downs in Hep3B cells in conditions corresponding to Figure 4A. ß-actin was used as loading control (A and B, left-hand graph). Inhibition of ROCK using Y-27632 was validated by the expression of P-Mypt-1. HSP90 was used as loading control (B, right-hand graph). **(C)** Protein expression of p190RhoGAP-A after its silencing in Hep3B (left-hand graph) and Huh7 (right-hand graph) cells, in conditions corresponding to Figure 4B. Error bars: SD of three or more independent experiments. Significance was determined with the Mann Whitney U test.

**Supplemental Figure 5: The combination of p190RhoGAP-A and Rnd3 silencing does not increase the entotic events in Hep3B and Huh7 cells.**

**(A)** Evaluation of the p190RhoGAP-A and Rnd3 protein expression after using siRNA by Western blot in Hep3B (Left panel) and Huh7 (Right panel) cells and the quantification of entotic events are represented in **(B)**. Error bars: SD of three or more independent experiments. Significance was determined with the Mann Whitney U test.

**Supplemental Figure 6: The silencing of E-cadherin alone increases the percentage of entotic events in Hep3B and Huh7 cells.**

**(A)** Validation of the inhibition of Rnd3 in the presence or absence of two siRNA targeting E-cadherin (#1 and #2) in Hep3B and Huh7 cell lines. **(B)** Evaluation of the entotic cells after inhibition of E-cadherin alone. Error bars: SD of three or more independent experiments. Significance was determined with the Mann Whitney U test.

**Supplemental Figure 7: Overexpression of E-Cadherin rescues entosis mediated by the loss of Rnd3.**

**(A-B)** Hep3B cells were transfected with indicated siRNA for 72h, and then transfected 48h with a plasmid encoding either GFP or E-Cadherin-GFP. Expression of Rnd3, GFP and Hsp90 (loading control) were monitored by Western-blot (A). Graphs present the quantification of E-cadherin-GFP, GFP and Rnd3 proteins (A) and of entotic cells (B) in all conditions. Error bars: SD of three independent experiments. Significance was determined with the Mann Whitney U test.

**Supplemental Figure 8: (A)** Functional interactome of the LAMP1 protein identified in the proteomic profile of entotic cells. Networks produced with the Ingenuity Pathways platform (IPA, QIAGEN). Proteins over-represented or under-represented in entotic cells were colored in red or green, respectively. **(B)** mRNA expression of *LAMP1* upon silencing of Rnd3. Hep3B cells were transfected by siRNA targeting Rnd3 (siRnd3#1 and siRnd3#3) or control siRNA (siCtrl) and transcriptome analysis was performed by RNAseq. The graph represents the normalized expression of the *LAMP1* gene (normalized read counts divided by median of transcripts length in kb). Significance was determined with the Mann Whitney U test. **(C)** Western blot and quantification of LAMP1 protein expression in Hep3B cells in conditions corresponding to Figure 4A and Supplemental Figure 4A. ß-actin was used as loading control. Error bars: SD of four independent experiments. Significance was determined with the Mann Whitney U test.

*Supplemental tables*

**Supplemental Table 1:** Results of the mass spectrometry analysis. List of proteins underrepresented in the entotic fraction

**Supplemental Table 2**: Results of the mass spectrometry analysis. List of proteins overrepresented in the entotic fraction

**Supplemental Table 3**: Results of the mass spectrometry analysis. GO analysis on proteins found underrepresented in the entotic fraction

**Supplemental Table 4**: Results of the mass spectrometry analysis. GO analysis on proteins found overrepresented in the entotic fraction

**Supplemental Table 5**: Sequences of siRNA used in this study

*Videos*

**Video 1**: **The video shows the entosis mechanism upon Rnd3 silencing in Hep3B cells.** Hep3B cell lines were transduced with H2B-GFP (yellow) and lifeact-mRuby (cyan) to mark the nucleus and the actin respectively. Cells were transfected with siRNA targeting Rnd3 and spinning disk microscopy analysis was done over 48 hours. Three stages were defined: i) cell contact, ii) internalization of the inner binuclear cell 21 hours after contact between cells and iii) beginning of the inner cell degradation 2 hours after internalization. The cell degradation takes almost 10 hours to be completed. Scale bar, 28 µm.
