# Supplementary material for: Loss of RND3/RHOE controls entosis through LAMP1 expression in hepatocellular carcinoma
Source: Cell Death Dis. 2024 Jan 13;15(1):46. doi: 10.1038/s41419-024-06420-3 (PMC10787830; doi:10.1038/s41419-024-06420-3)

Page Ruler Plus Prestained Protein Ladder 10 to 250 kDa #26619
Used for all western blot experiment

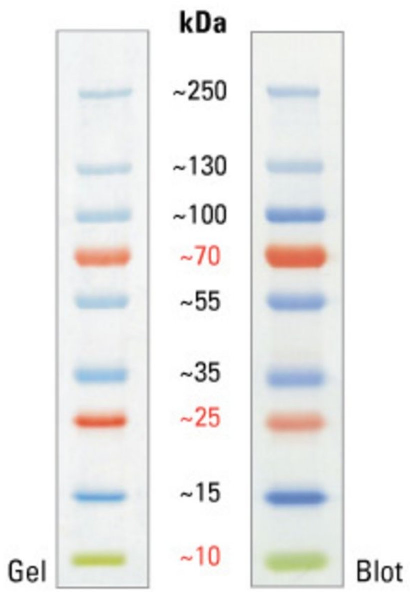

Figure 2

A

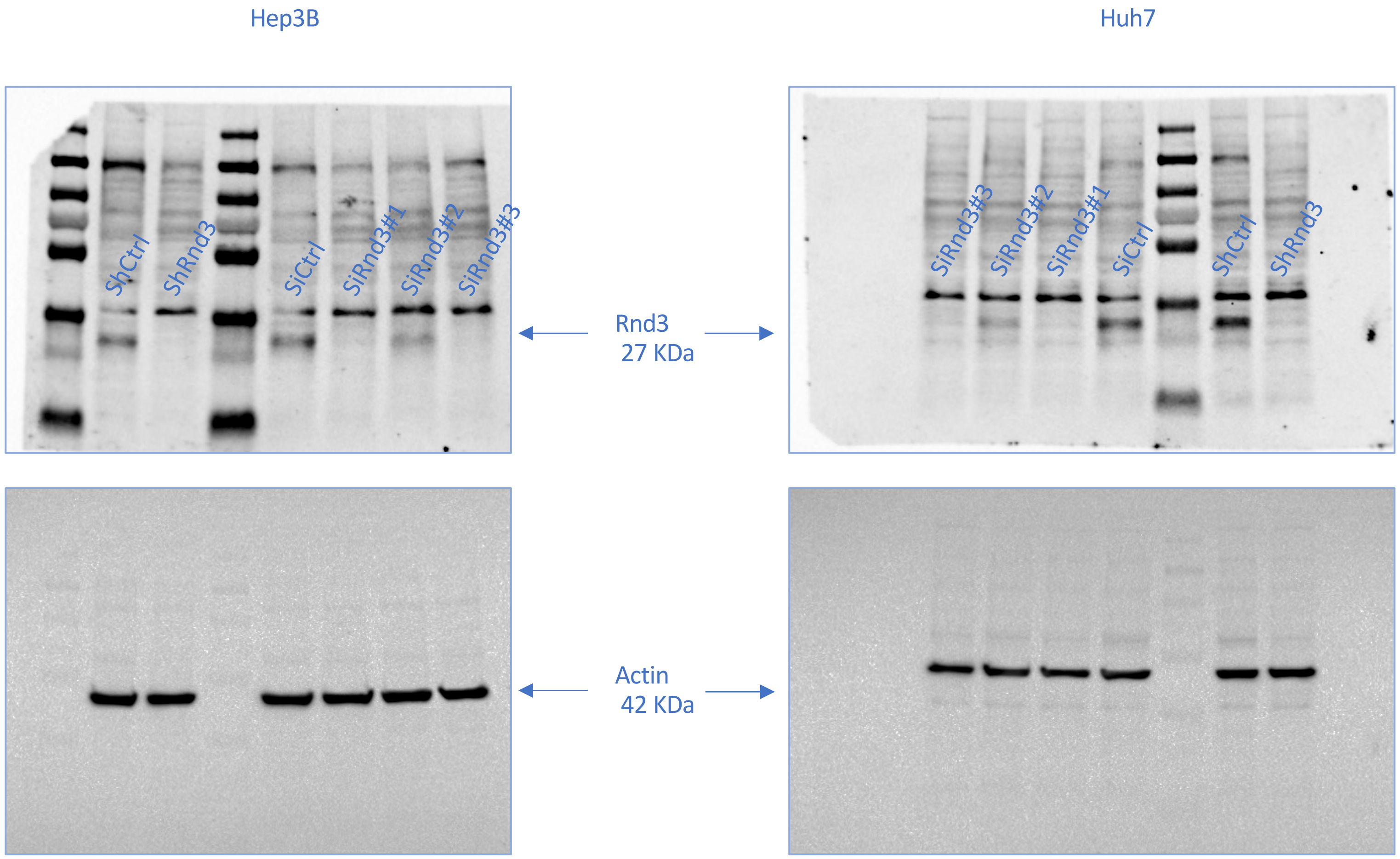

Hep3B
Huh7
ShCtrl
ShRnd3
SiCtrl
SiRnd3#1
SiRnd3#2
SiRnd3#3
SiRnd3#3
SiRnd3#2
SiRnd3#1
SiCtrl
ShCtrl
ShRnd3
Rnd3
27 KDa
Actin
42 KDa

Figure 4

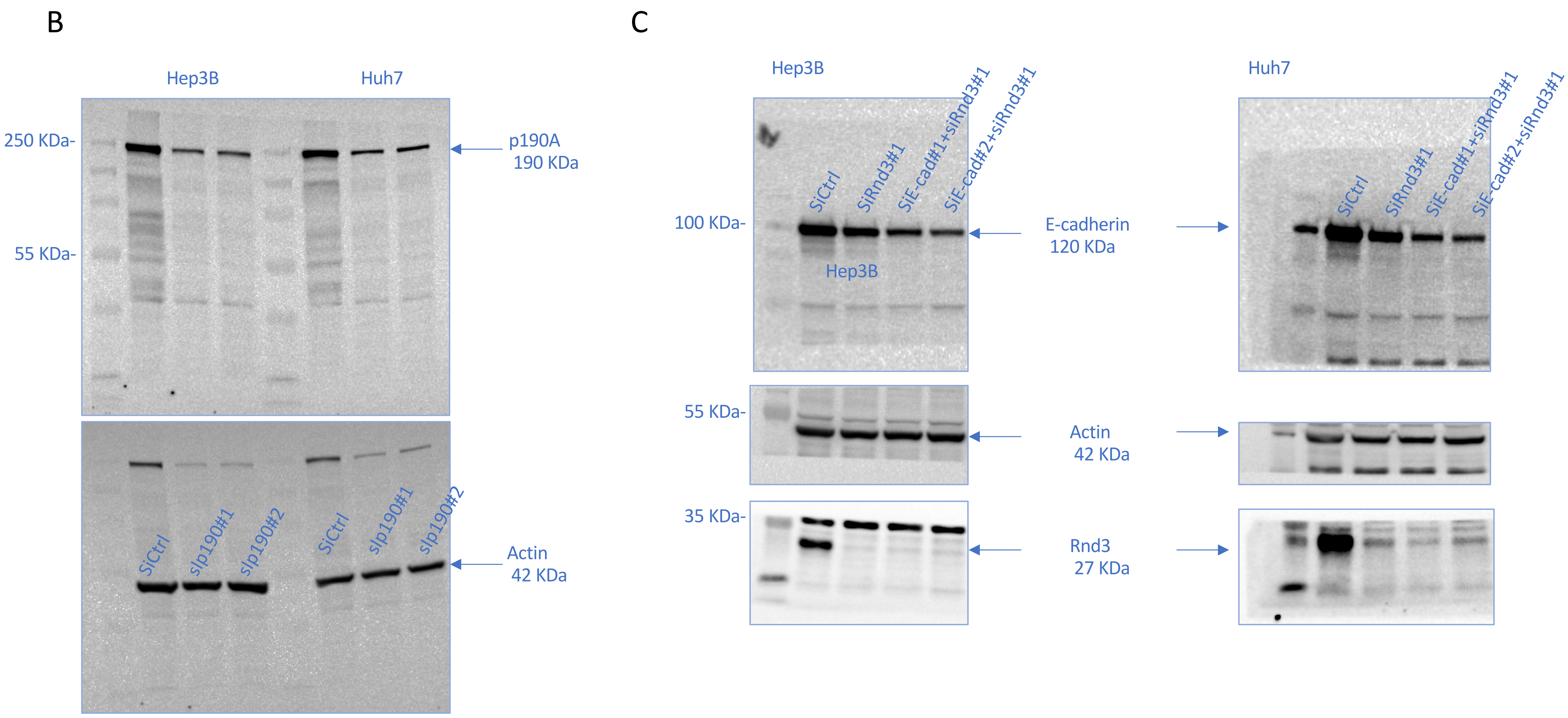
B
Hep3B
Huh7
250 KDa-
55 KDa-
p190A
190 KDa
SiCtrl
sIp190#1
sIp190#2
SiCtrl
sIp190#1
sIp190#2
Actin
42 KDa
C
Hep3B
Huh7
SiCtrl
SiRnd3#1
SiE-cad#1+siRnd3#1
SiE-cad#2+siRnd3#1
100 KDa-
E-cadherin
120 KDa
Hep3B
55 KDa-
Actin
42 KDa
35 KDa-
Rnd3
27 KDa
SiCtrl
SiRnd3#1
SiE-cad#1+siRnd3#1
SiE-cad#2+siRnd3#1

Figure 5

C

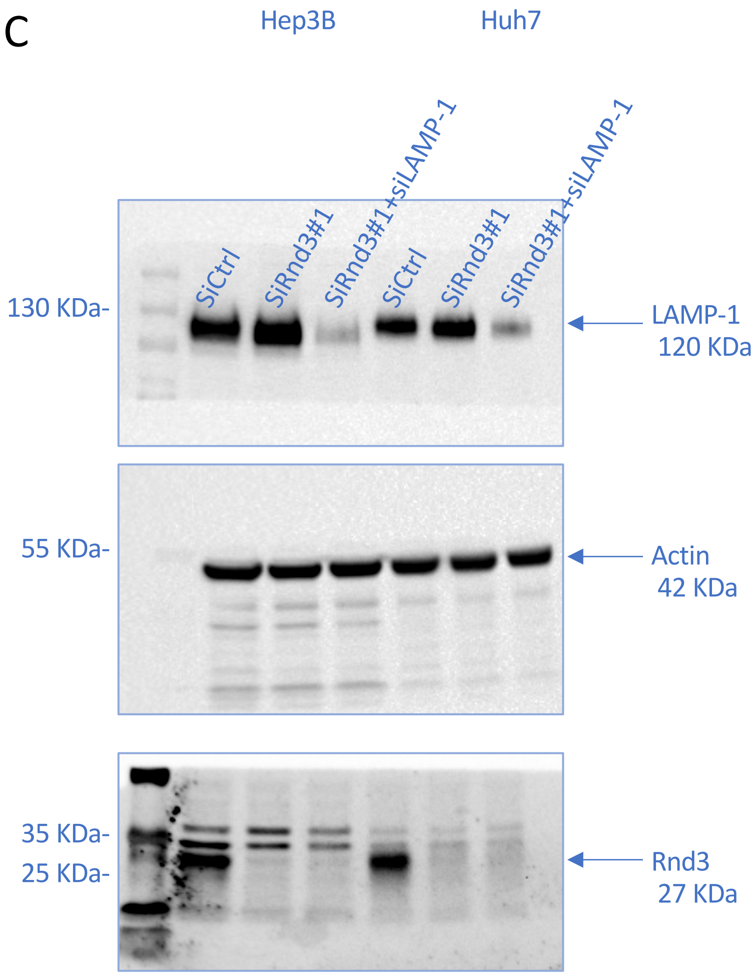

Hep3B
Huh7
SiCtrl
SiRnd3#1
SiRnd3#1+siLAMP-1
SiCtrl
SiRnd3#1
SiRnd3#1+siLAMP-1
130 KDa-
LAMP-1
120 KDa
55 KDa-
Actin
42 KDa
35 KDa-
25 KDa-
Rnd3
27 KDa

Supplemental Figure 1

C

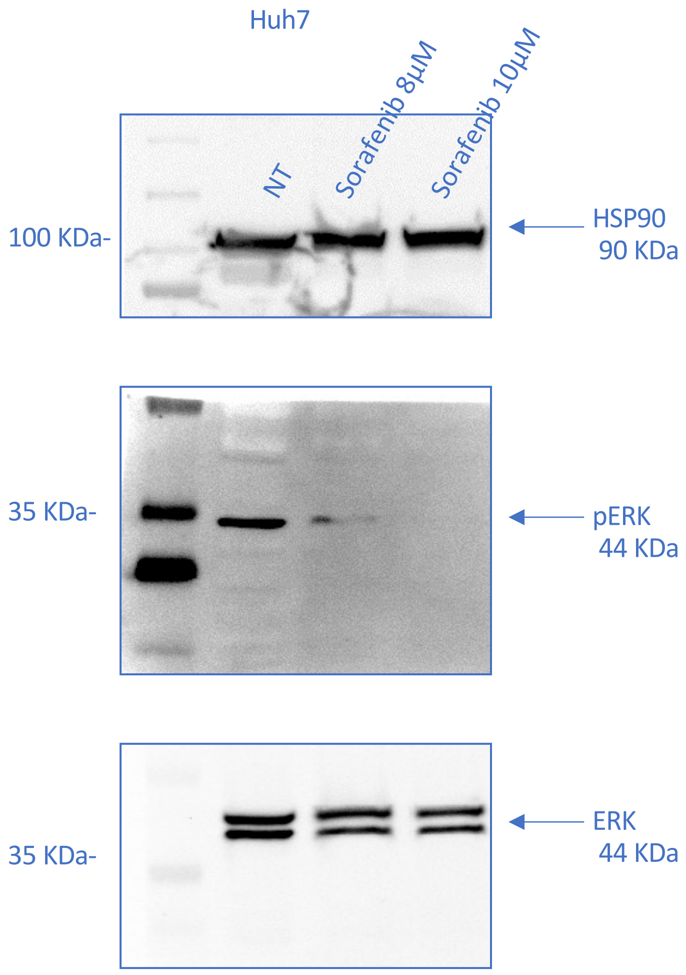

Supplemental Figure 2

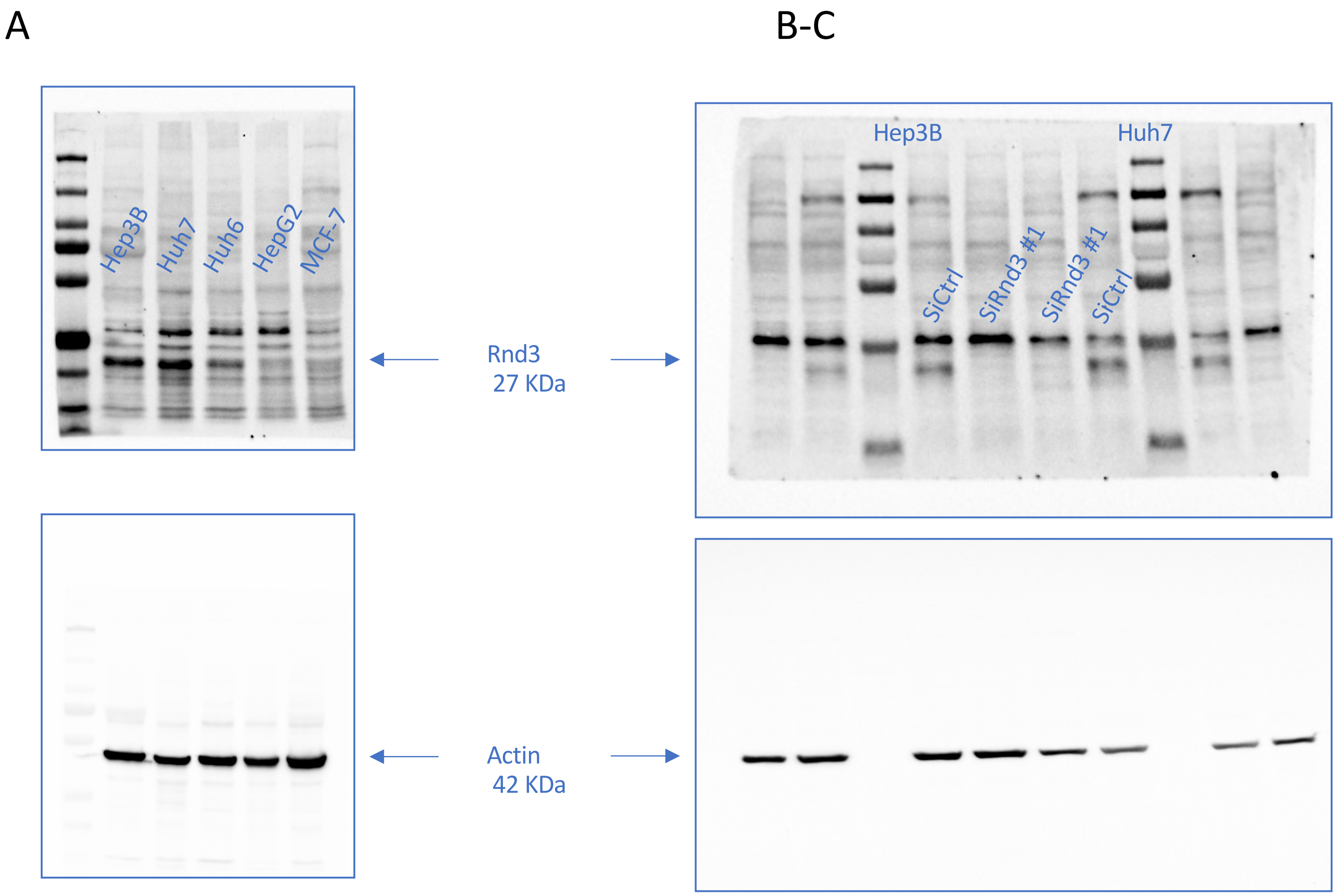

Supplemental Figure 3

B

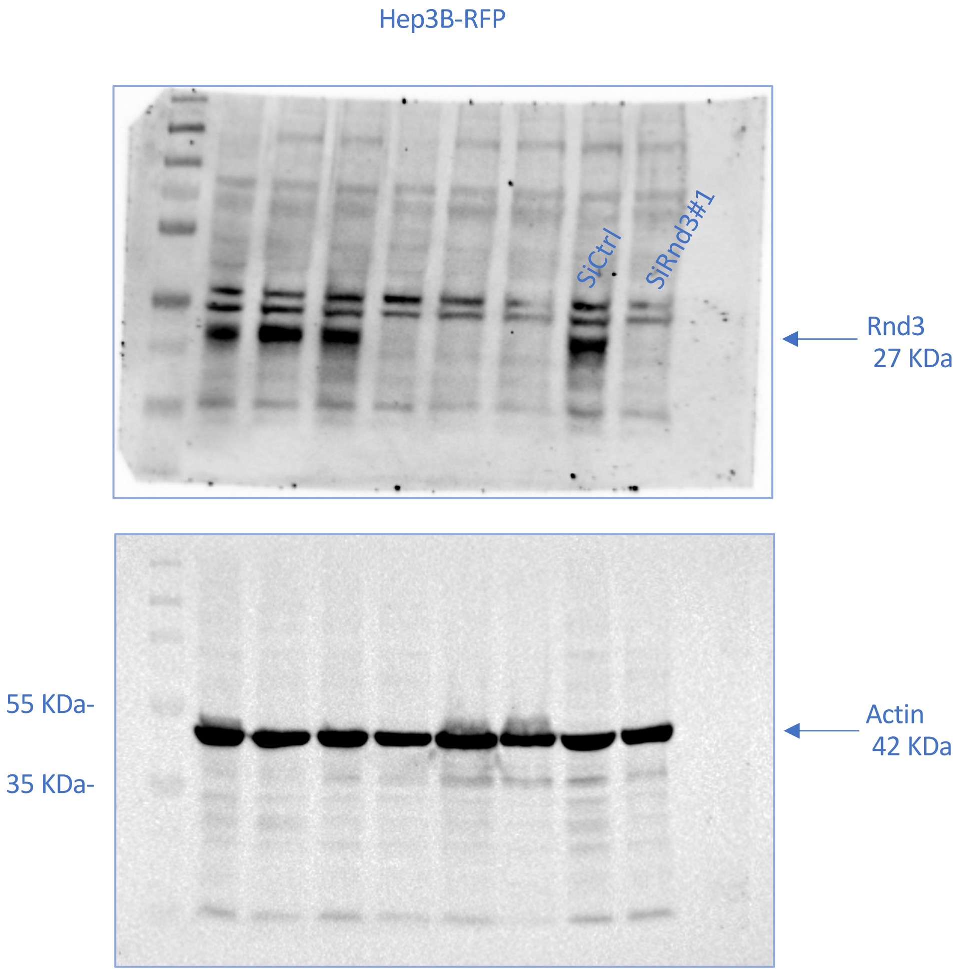

Supplemental Figure 4

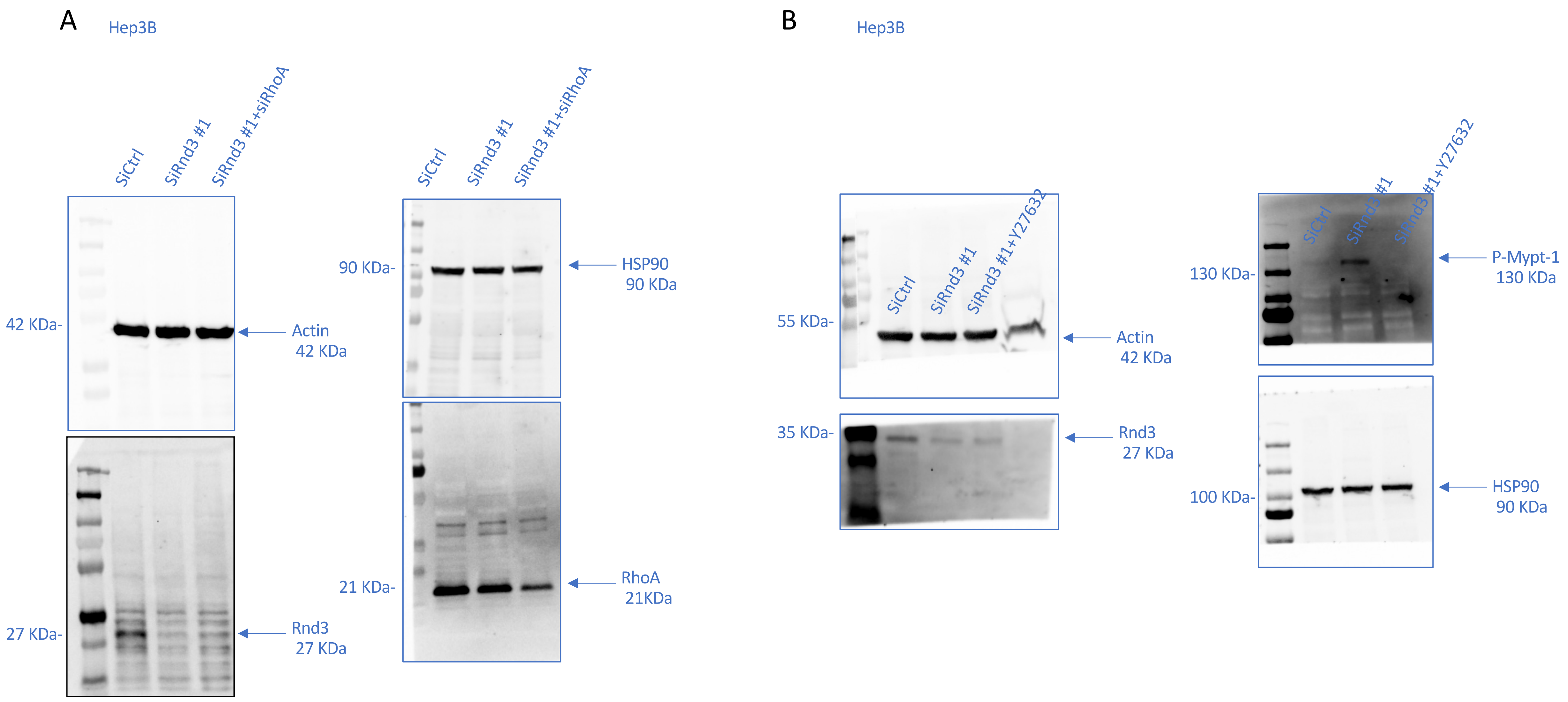

Supplemental Figure 5

A

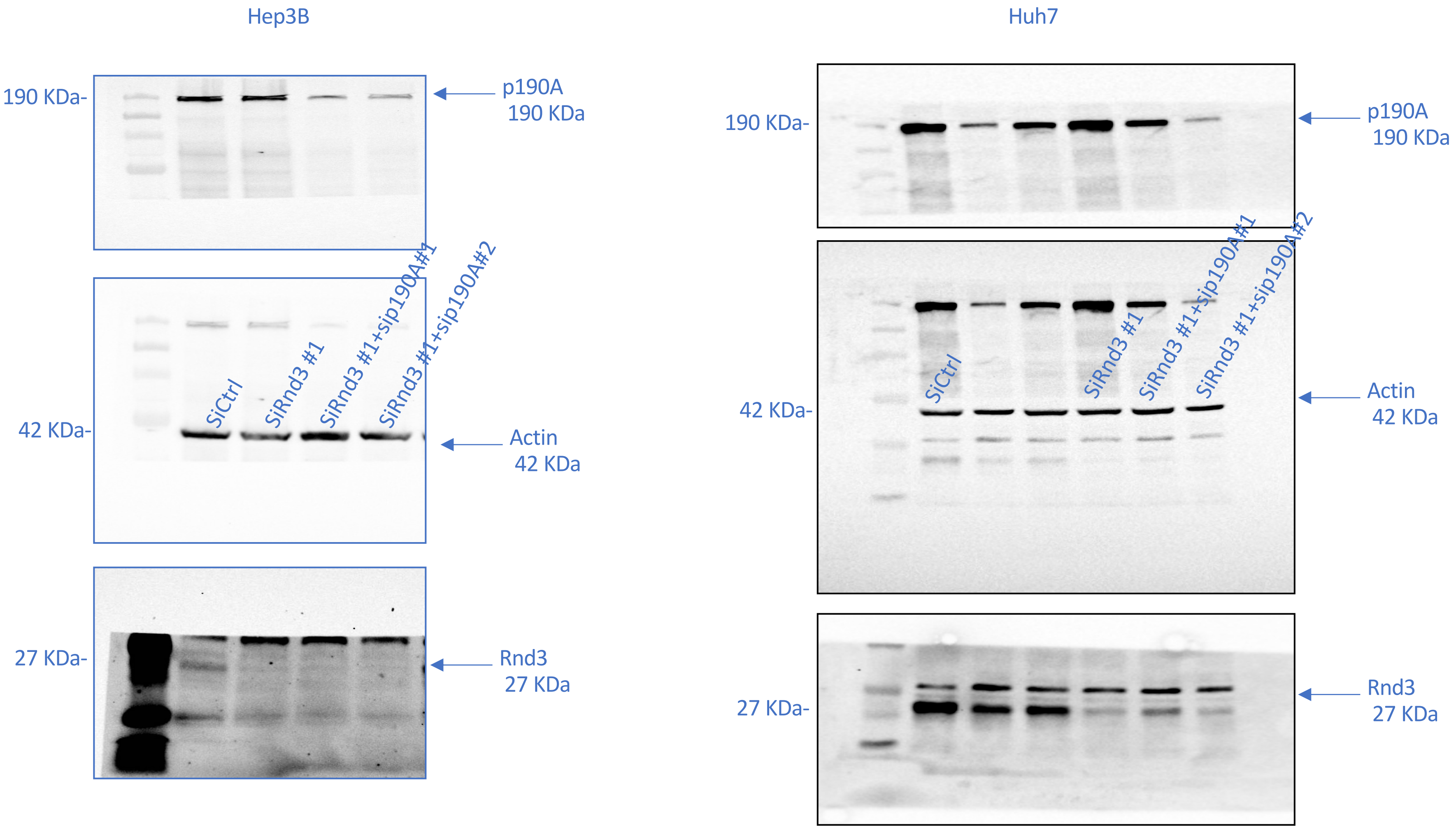

Supplemental Figure 6

B

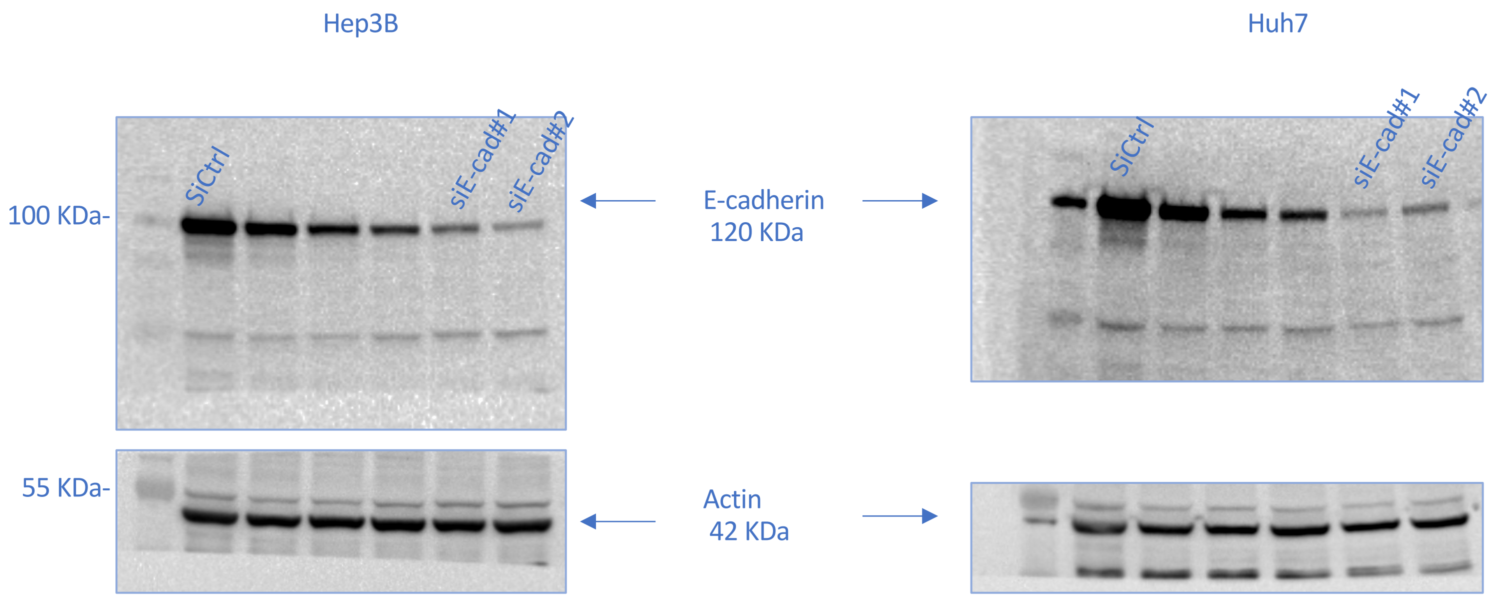

Supplemental Figure 7

A

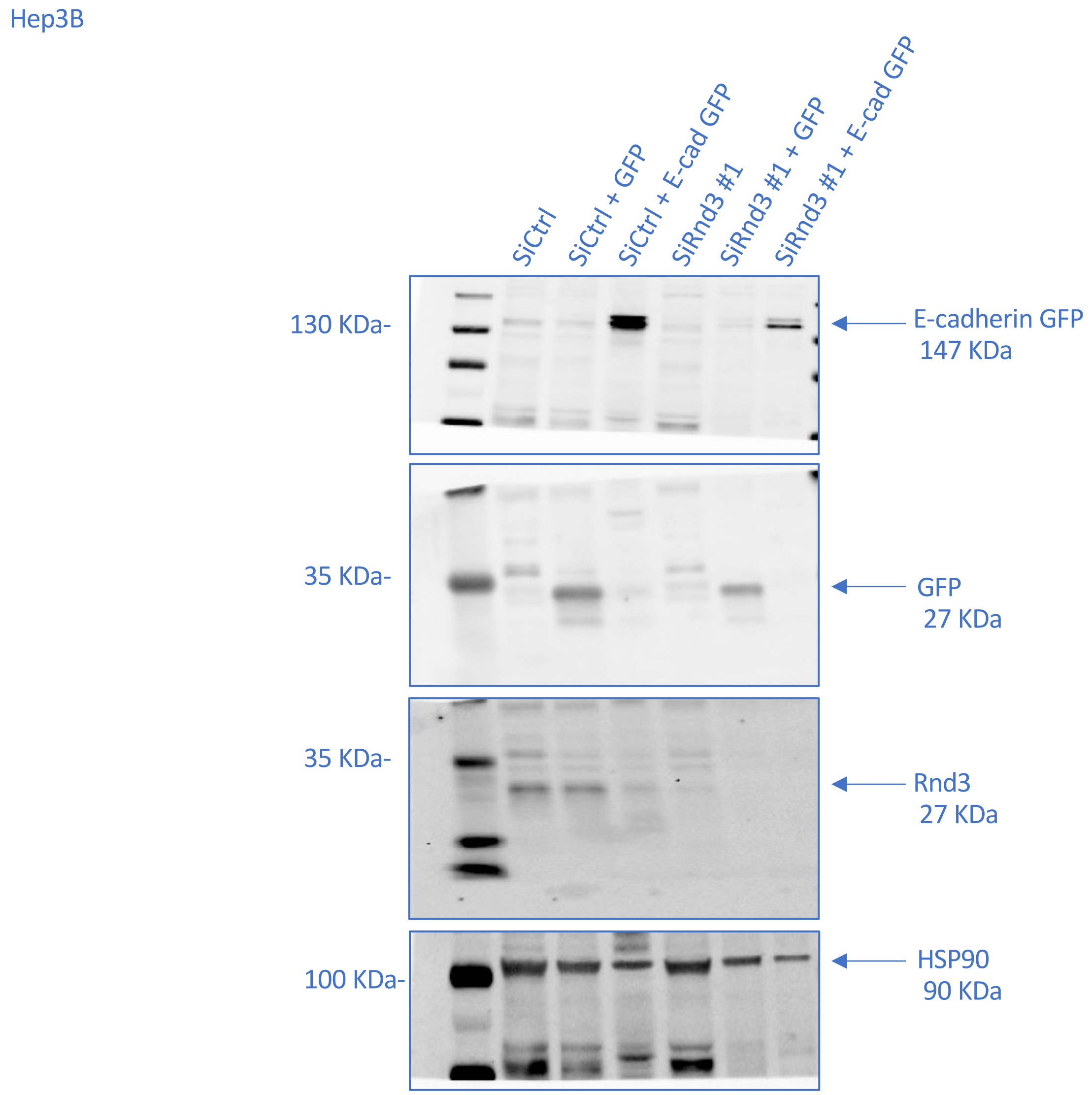

Supplemental Figure 8

C

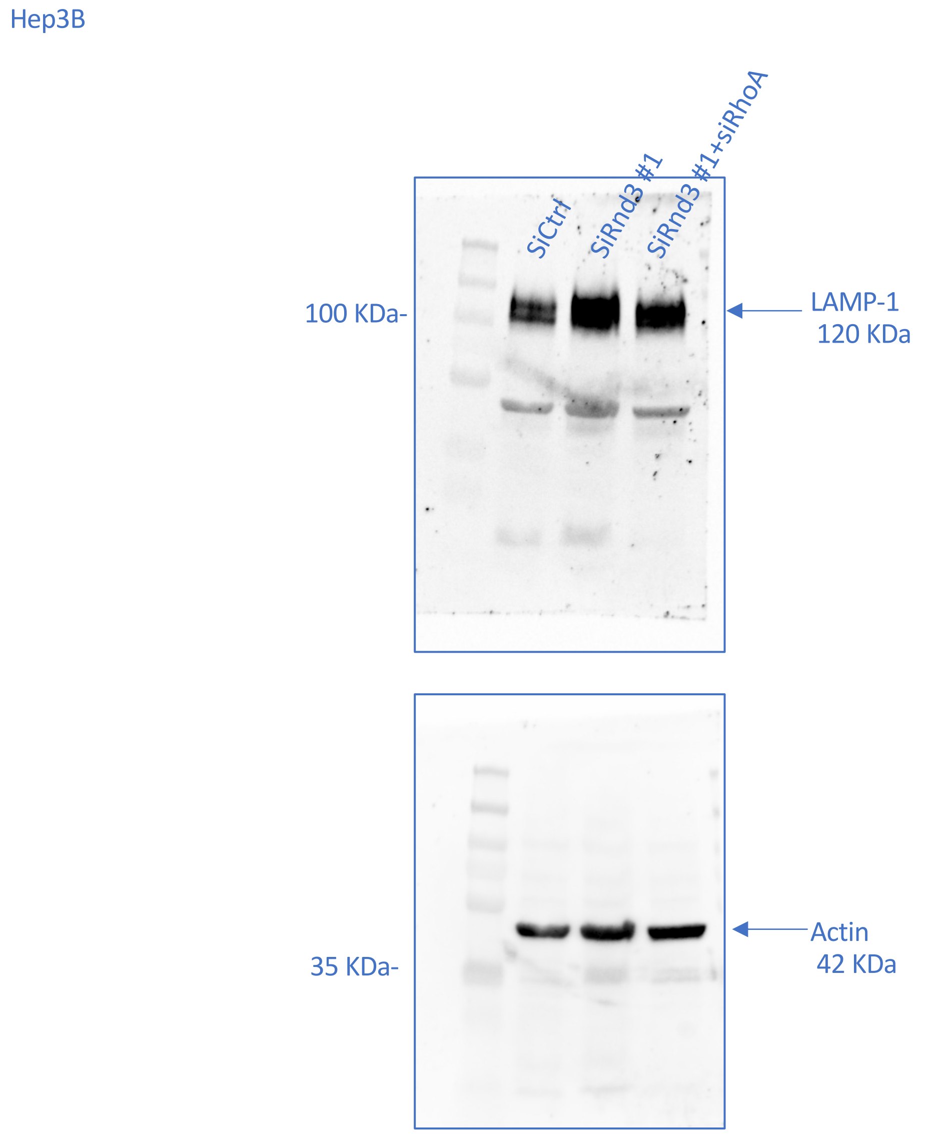

Supplement: Supplementary file 9 — Original Data File [file 41419_2024_6420_MOESM9_ESM.pdf]
